# Ataxia telangiectasia derived iPS cells show preserved x-ray sensitivity and decreased chromosomal instability

**DOI:** 10.1038/srep05421

**Published:** 2014-06-27

**Authors:** Yoshihiro Fukawatase, Masashi Toyoda, Kohji Okamura, Ken-ichi Nakamura, Kazuhiko Nakabayashi, Shuji Takada, Mayu Yamazaki-Inoue, Akira Masuda, Michiyo Nasu, Kenichiro Hata, Kazunori Hanaoka, Akon Higuchi, Kaiyo Takubo, Akihiro Umezawa

**Affiliations:** 1 Department of Reproductive Biology, National Research Institute for Child Health and Development, Tokyo, 157-8535, Japan; 2 Department of Systems BioMedicine, National Research Institute for Child Health and Development, Tokyo, 157-8535, Japan; 3 Department of Maternal-Fetal Biology, National Research Institute for Child Health and Development, Tokyo, 157-8535, Japan; 4 School of BioMedical Science, Tokyo Medical and Dental University, Tokyo, 113-0034, Japan; 5 Department of Research Team for Geriatric Medicine, Tokyo Metropolitan Institute of Gerontology, Tokyo, 173-0015, Japan; 6 Department of Research Team for Geriatric Pathology, Tokyo Metropolitan Institute of Gerontology, Tokyo, 173-0015, Japan; 7 Department of BioSciences, Kitasato University School of Science, Kanagawa, 252-0373, Japan; 8 Department of Chemical and Materials Engineering, National Central University, Taoyuan, 32001, Taiwan; 9 College of Science, King Saud University, Riyadh, 11451, Saudi Arabia

## Abstract

Ataxia telangiectasia is a neurodegenerative inherited disease with chromosomal instability and hypersensitivity to ionizing radiation. iPS cells lacking ATM (AT-iPS cells) exhibited hypersensitivity to X-ray irradiation, one of the characteristics of the disease. While parental ataxia telangiectasia cells exhibited significant chromosomal abnormalities, AT-iPS cells did not show any chromosomal instability *in vitro* for at least 80 passages (560 days). Whole exome analysis also showed a comparable nucleotide substitution rate in AT-iPS cells. Taken together, these data show that ATM is involved in protection from irradiation-induced cell death.

The technology to generate human induced pluripotent stem cells (iPS cells) has impacted various medical fields, such as clinical applications and drug discovery, as well as basic biological science on reprogramming of differentiated cells^1^,^2^. The most recent attention has been placed on their potential use in cell-based transplantation. Using *in vitro* differentiation, iPS cells, like embryonic stem cells (ES cells), can provide an unlimited source of useful cell types for transplantation. The use of iPS cells in clinical application and research has been largely welcomed by society because use of these cells avoids the substantial ethical concern of cellular origin that plagues ES cells. The fact that the cells are autologous for patients could be another advantage in transplantation. Soon after human iPS cell technology was introduced, researchers also began to realize an additional and possibly greater value for the technology as a system to model human diseases^3^. Since iPS cells can be generated from skin biopsies or blood samples, retain all the genomic information from the original patients, and can be differentiated *in vitro* into cell types which are not easily accessible in patients, iPS cells can be utilized to study how genetic aberrancies in the patient manifest in target cells *in vitro*.

Ataxia telangiectasia (AT) is a rare neurodegenerative inherited disease characterized by early-onset progressive cerebellar ataxia, telangiectasias of the eyes and skin, immunodeficiency, chromosomal instability, hypersensitivity to ionizing radiation, and increased risk of cancer^4^. AT is caused by a defect in the *ATM* gene, which is responsible for recognizing and correcting DNA damage, and for destroying the cells when the errors cannot be corrected. One feature of ATM protein is its rapid increase in kinase activity immediately after double-strand DNA break formation^5^. The phenotypic manifestation of AT is due to the broad range of phosphorylation of substrates for the ATM kinase, involving DNA repair, apoptosis, G_1_/S, intra-S checkpoint and G_2_/M checkpoints, gene regulation, translation initiation, and telomere maintenance^6^. Therefore, a defect in *ATM* has severe consequences, and may lead to tumor formation. For example, the increased risk for breast cancer in AT patients implicates the involvement of ATM in the interaction and phosphorylation of BRCA1 and its associated proteins following DNA damage^7^.

Though the molecular basis of AT, such as a defect in the *ATM* gene and the effect that has on the broad range of substrates for the ATM kinase has been well established, the linkage between the loss of ATM function and various clinical outcomes remain still unclear. *Atm*-deficient mice have been created to recapitulate the human disease and then characterized to understand the relationship between the AT phenotype and the pleiotropic function of *Atm*^8^. Mice homozygous for *Atm* disruption show growth retardation, neurologic dysfunction, immunologic abnormalities, lymphoreticular malignancies, chromosomal instability, and extreme sensitivity to ionizing radiation. However, oculocutaneous telangiectasias and remarkable histological evidence of neuronal degeneration, which are characteristics of human AT patients, have not been seen in these mice. The mouse model for AT is, therefore, very useful, but limited for understanding the human disease.

Mouse iPS cells from tail-tip fibroblasts of *Atm*-deficient mice have been reported^9^,^10^. Reprogramming efficiency is greatly reduced in the fibroblasts of *Atm*-deficient mice. Likewise, *ATM*-deficient human pluripotent stem cells, i.e. ES cells and iPS cells, have successfully been established by disrupting *ATM* gene^11^ and from patients with ataxia telangiectasia^12^,^13^, respectively. These pluripotent stem cells exhibit disease-specific characteristics such as radiosensitivity and cell cycle checkpoint defects, and therefore serve disease model cells for clarification of pathogenic mechanism and screening novel compounds to treat the disease. The AT-iPS cell platform was indeed used to screen low-molecular compounds^12^.

In the present study, we attempted to generate iPS cells from fibroblasts of ataxia telangiectasia (AT-iPS cells), and successfully established the cells from the fibroblasts of AT patients. The reprogramming efficiency was very low as previously reported in the establishment of murine ATM-KO iPS cells. Human AT-iPS cells exhibited hypersensitivity to X-ray irradiation. Unexpectedly, the human AT-iPS cells did not show any chromosomal instability *in vitro*, i.e. maintenance of intact chromosomes lasted for at least 80 passages (560 days). These results indicate that the established human AT-iPS cells may be useful for the exploration of the mechanism of reprogramming, for clarifying the pathogenesis of AT, and for facilitating novel therapeutic interventions of the human disease. The possible mechanisms for the low reprogramming efficiency and for stable maintenance of their chromosome in the AT-iPS cells will also be discussed.

## Results

### Generation of iPS cells from human AT cells

**Figure 1:**
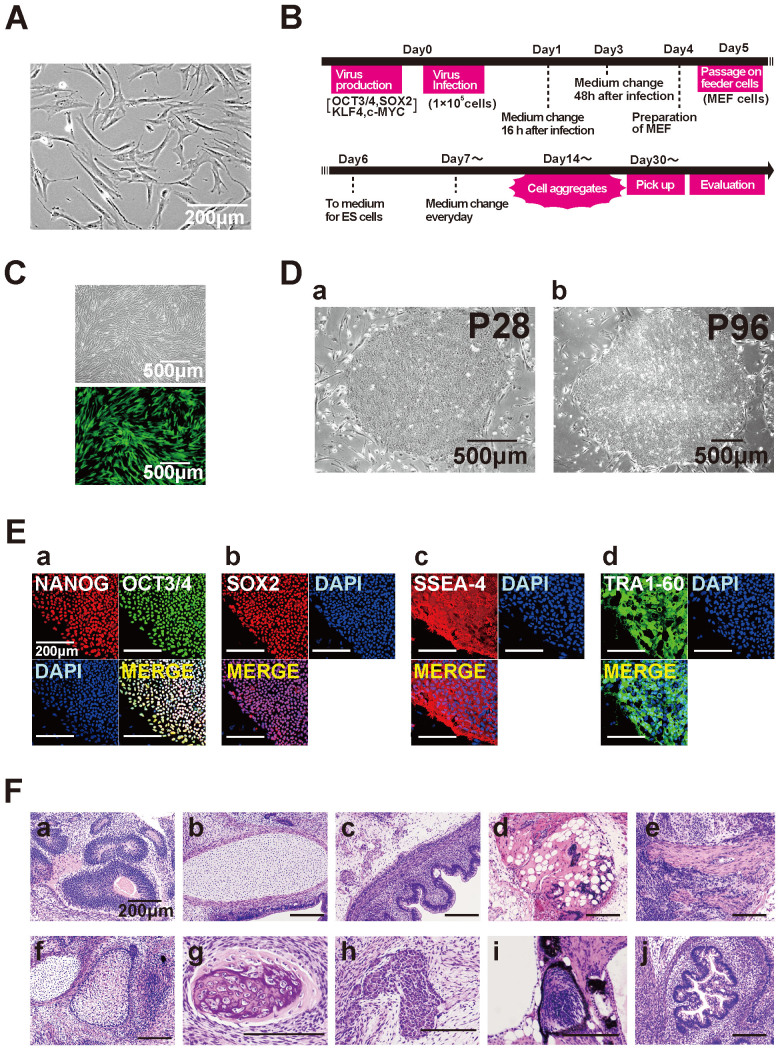
Generation of iPS cells from ataxia telangiectasia-derived cells. (A). Phase-contrast image of AT1OS ataxia telangiectasia-derived cells. (B). Protocol for iPS cell generation. (C). Infection efficiency as assessed by retrovirus carrying the EGFP gene. (D). Phase-contrast microphotographs of AT-iPS cell clones at passages 28 and 96. (E). Immunocytochemical analysis of AT-iPS cells using antibodies to NANOG (a), OCT3/4 (a), SOX2 (b), SSEA-4 (c), and TRA-1-60 (d). (F). Histology of teratoma generated by AT-iPS cells. (a): ectodermal glia and neuroepithelium, (b): cartilage, (c): intestinal epithelium, (d): adipose tissue, (e): smooth muscle, (f): epidermis, (g): bone, (h): hepatocytes, (i): retina, (j): intestine.

It has been reported that fibroblasts from *Atm*-deficient mice show remarkably low reprogramming efficiency compared to normal fibroblasts. Thus, we examined whether iPS cells could be efficiently generated from human cells having a mutation in the *ATM* gene (AT1OS cells, Figure 1A) by using the vesicular stomatitis virus G glycoprotein (VSV-G) retroviral transduction system (Figure 1B). By using this system, the transduction efficiency was 53.8% ± 11.9% (mean ± standard deviation) as estimated by enhanced green fluorescent protein (EGFP) expression (Figure 1C). Southern blot analysis with cDNA probes for each of four transgenes (*OCT-3/4, SOX2, KLF-4*, and *c-MYC*) confirmed that each clone had chromosomal integration of the exogenously infected genes (Supplemental Figure S1). When the reprogramming factors OCT3/4, SOX2, KLF4 and c-MYC were introduced in 2.0 × 10^5^ AT1OS cells, only 10 iPS colonies were successfully generated. We compared the reprogramming efficiency of AT-iPS cells with that of MRC5-iPS cells that were generated by the same VSV-G retrovirus construct and protocol. The efficiency of AT-iPS cell colony generation (0.005%) was approximately 1/100, compared with that of MRC5-iPS cell generation (0.5%). Morphological characteristics of AT-iPS cells were similar to those of other intact iPS cells and ES cells (Figure 1D). Immunohistochemical analyses demonstrated that expression of the pluripotent cell-specific nuclear proteins, OCT3/4, SOX2 and NANOG, and the keratan sulfate proteoglycan TRA-1-60 (Figure 1E, Supplemental Figure S2) was consistent with the profile observed in hES cells. Hierarchical clustering analysis and principle component analysis of gene chip analysis data revealed that AT-iPS cells were grouped into the same category as MRC5-iPS cells, but not grouped into the ES cell category and parental cell category, regardless of gene set: all genes, neural genes, DNA-damage genes, and cell cycle-related genes (Supplemental Figure S3).

### Teratoma formation of AT-iPS cells

To address whether the AT-iPS cells have competence to differentiate into specific tissues, teratoma formation was performed by implantation of AT-iPS cells at the subcutaneous tissue (1.0 × 10^7^ cells/site) of immunodeficient NOD/SCID mice. Four independent AT-iPS cell clones induced teratomas within 6–10 weeks after implantation. Histological analysis of paraffin-embedded sections demonstrated that the three primary germ layers were generated as shown by the presence of ectodermal glia and neuroepithelium, mesodermal muscle and cartilage, endodermal ciliated epithelium morphologically in the teratoma (Figure 1F). Thus, all AT-iPS cell clones examined had potential for multi-lineage differentiation *in vivo*.

### Characterization of AT-iPS cells

**Figure 2:**
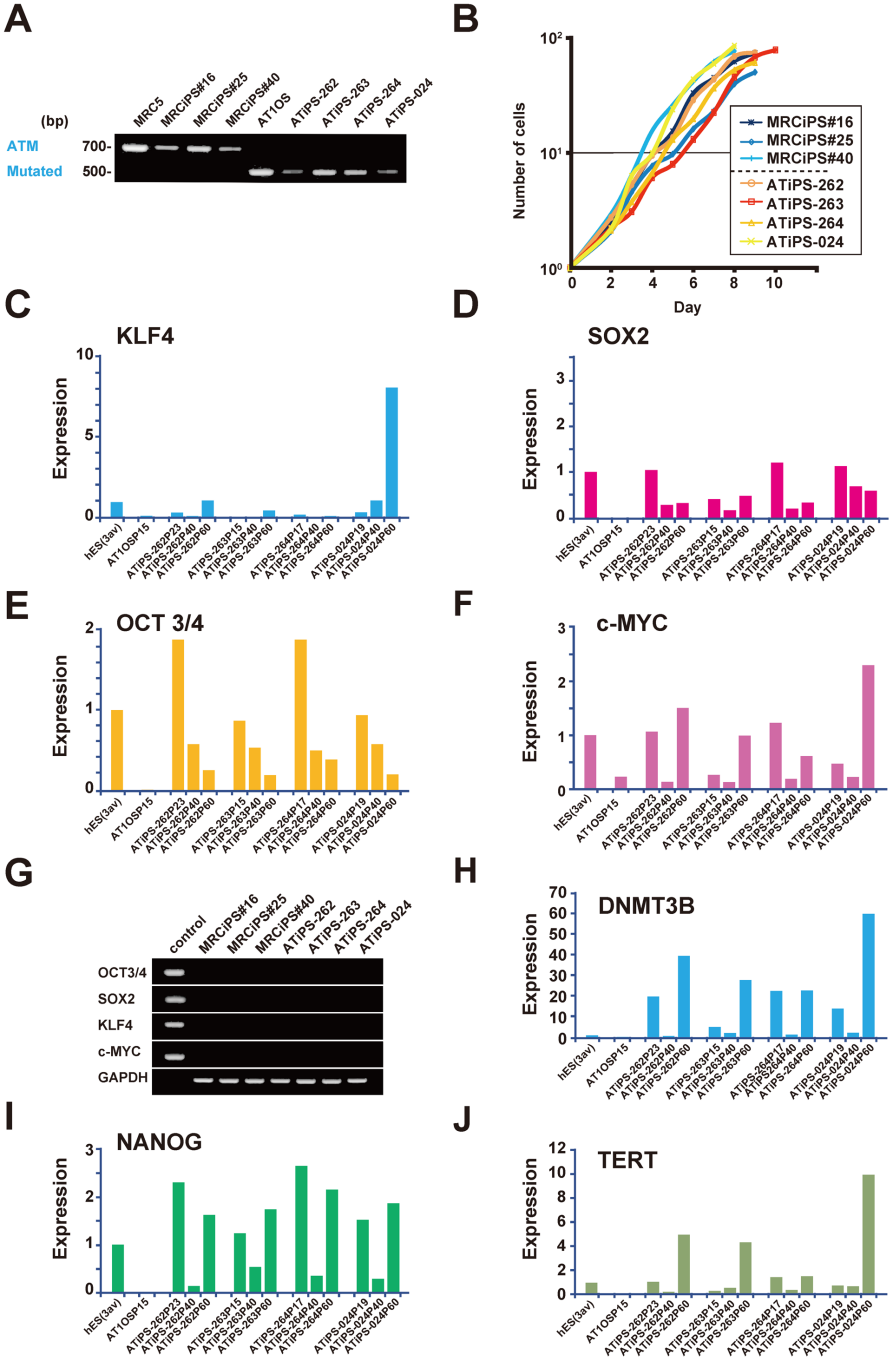
Expression of the endogenous genes and the transgenes. (A). Expression of intact and mutated *ATM* gene in MRC5-iPS and AT-iPS cells. (B). Growth curves of AT-iPS and MRC5-iPS cells. Cell number was counted on the indicated day after cells (10^5^ cells/dish) were seeded on matrigel-coated 6-well plates. (C). Expression of the endogenous *KLF4* gene. (D). Expression of the endogenous *SOX2* gene. (E). Expression of the endogenous *OCT-3/4* gene. (F). Expression of the endogenous *c-MYC* gene. (G). Expression of the *OCT-3/4, SOX2, KLF4*, and *c-MYC* transgenes in each iPS cell at passage 10 (more than 30 population doublings). (H). Expression of the *DNMT-3B* gene. (I). Expression of the *NANOG* gene. (J). Expression of the *TERT* gene.

We examined the expression of the mutated *ATM* gene in AT-iPS cells by RT-PCR for amplifying the sequence including exon 31 of the *ATM* gene to confirm that the established cells were AT-derived (Figure 2A, Supplemental Table S1). AT-iPS cells clearly retained expression of the mutated *ATM* gene that had a deletion of 165 bp corresponding to the deletion of exon 31, showing that these cell clones were actually AT1OS derived. We also performed protein blot analysis on AT-iPS and MRC5-iPS cells (Supplemental Figure S4). ATM was detected at the protein level in MRC5-iPS cells, but not in AT-iPS cells (Supplemental Figure S4A, B). p53 was expressed at a similar level in AT-iPS and MRC5-iPS cells, and phosphorylation of p53 on serine-15 was similar in AT-iPS and MRC5-iPS cells (Supplemental Figure S4C, D).

The proliferative capacity of four AT-iPS cell clones was measured and compared with that of three MRC5-iPS cell clones (Figure 2B). No significant differences in proliferation rates were detected between the AT-iPS cell clones and the MRC5-iPS cell clones. Continuous observation through 18 passages revealed that AT-iPS cells continued to expand at a rate similar to MRC5-iPS cells, and could be cultured for more than 20 passages. Neither cessation of cell proliferation like senescence nor apoptosis/cell death was detected during cultivation through 20 passages.

### Stem cell-associated gene expression in AT-iPS cells

The expression profiles of stem cell-associated genes were examined with qualitative RT-PCR to confirm the iPS cell characteristics of the established cell clones. The expression of the endogenous reprogramming factor genes (*KLF4*, *SOX2*, *OCT3/4*, and *c-MYC*) were undetectable or very low in the parental AT1OS cells, but were all activated in AT-iPS cells (Figure 2C to F). While the transgenes were fully silenced in AT-iPS cells (Figure 2G), expression of pluripotent cell-specific genes, such as *DNMT3B, NANOG*, and *TERT*, were activated in all AT-iPS cell clones to a similar extent of those in control hES cells and MRC5-iPS cells (Figure 2H to J, Supplemental Figure S5).

### Karyotypic analysis of AT-iPS cells during cultivation

**Figure 3:**
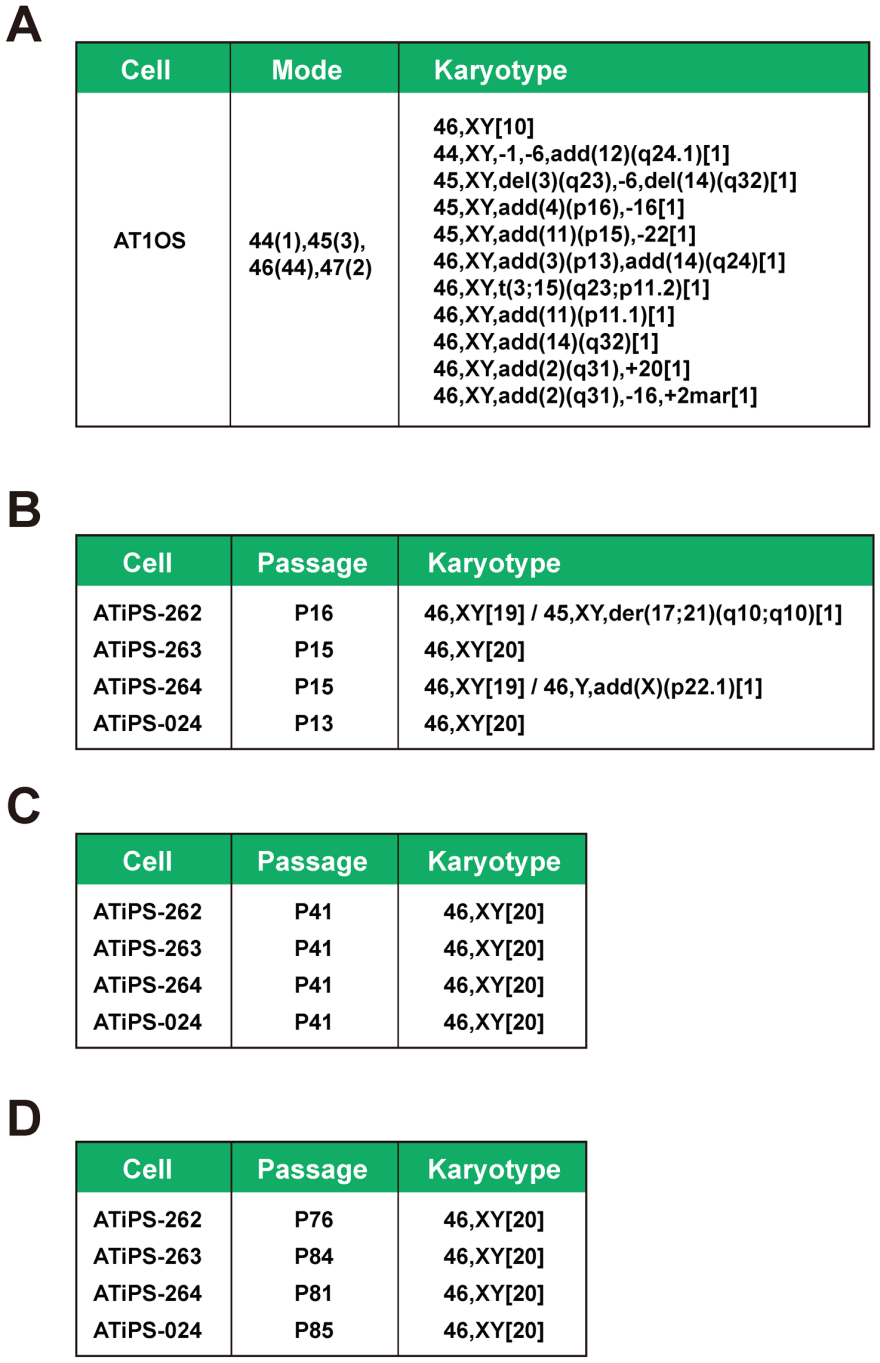
Karyotypes of AT-iPS cells and their parental cells after long-term cultivation. (A). Karyotypic analysis of AT1OS parental cells. (B). Karyotypic analyses of AT-iPS cell clones at Passage 13–16. (C). Karyotypic analyses of AT-iPS cell clones at Passage 41. (D). Karyotypic analyses of AT-iPS cell clones at Passage 76–85. For karyotypic analysis, 20 cells were analyzed and the number of cells with the indicated karyotype was shown in brackets.

AT is a chromosome instability syndrome. The patients' cells frequently show chromosomal aberrations such as spontaneous chromatid/chromosome breaks, triradials, quadriradials and telomeric associations as well as numerical anomalies. In general, fibroblastic cell lines derived from AT patients accumulate chromosomal aberrations with an increase in passage number. Therefore, we performed karyotypic analyses of the AT1OS parental cells and AT-iPS cell clones at various passages (until 41 passages for more than 10 months). Parental AT1OS cells frequently exhibited chromosomal abnormalities, such as deletion, addition and translocation (Figure 3A). In contrast, most cells of the four AT-iPS cell clones had an intact karyotype at passage 13 to 16 (Figure 3B–D). Even after a long cultivation period (passage 41), karyotypes of all cells of the four AT-iPS cell clones remained intact. Morphological characteristics of AT-iPS cell colonies, i.e. the growth of flat and aggregated colonies, did not significantly change even at passage 96 (Figure 1D). Also, AT-iPS cells retained high alkaline phosphatase activity and teratoma formation after a long-term cultivation (Supplemental Figure S6).

### Elongated telomere length in AT-iPS cells

Telomere lengths in AT1OS cells and AT-iPS cells were measured (Table 1). TIG-1 at 34 population doublings served as a telomere length standard (6.91 kbp). The established ATiPS-262, -264, and -024 cells had 13.14, 15.64, and 16.54 kbp in telomere length, while the parental AT1OS cells were 4.13 kbp. The results clearly show that AT-iPS cells gain elongated telomeres after iPS cell generation.

### Genomic alteration during AT-iPS cell cultivation

**Figure 4:**
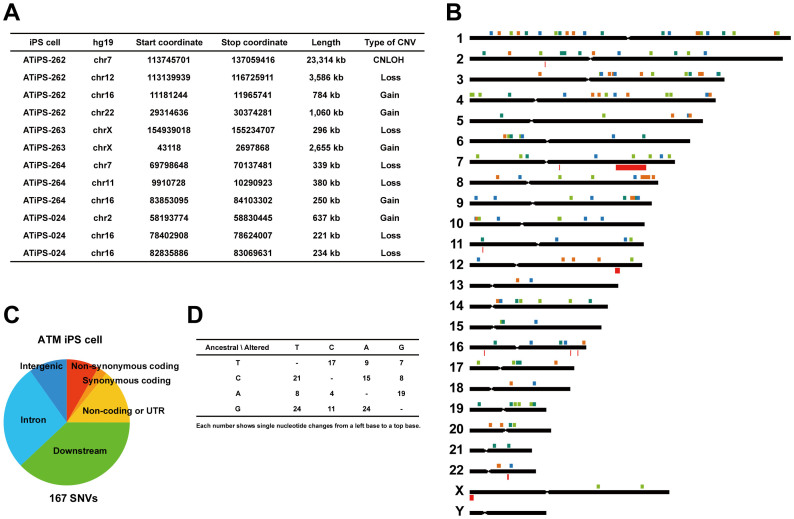
Genomic alterations in AT-iPS cell cultivation. (A). Structural alterations identified by a comparison between parental AT1OS cells and AT-iPS cells. (B). Genome-wide distribution of SNVs identified in the AT-iPS cells. Filled squares above a chromosomal bar stand for genomic position of each SNV. Orange, yellowish green, green, and blue squares indicate alteration events occurred in the ATiPS-262, ATiPS-263, ATiPS-264, and ATiPS-024 iPS cells, respectively. A 23-Mb copy-neutral LOH region on chromosome 7 (ATiPS-262), a 3.6-Mb deletion on chromosome 12 (ATiPS-262), and a 0.23-Mb deletion on chromosome 16 (ATiPS-024) involved 2, 37, and 1 nucleotide mismatches detected during the course of our exome analysis, respectively. (C). Pie chart illustrating the ratio of protein-coding and non-coding sequences. Our exome analysis targeted 93,907,534 bases in the genome, and 30,331,579 bases within them are considered coding sequences, start or stop codons. Covering large part of non-coding sequences was one of features of the present exome study. Note that numbers of non-synonymous coding bases are larger than those of synonymous coding bases in the AT-iPS cells. Annotations were performed using SnpEff 3.1 (http://snpeff.sourceforge.net/SnpEff_manual.html). For example, if a variation was positioned within 5 kb from the 3′ end of a gene, it was annotated as “downstream.” (D). Single nucleotide change patterns observed in AT-iPS cells.

Because AT1OS cells exhibited considerable chromosomal abnormalities *in vitro*, we performed a structural alteration analysis using a SNP genotyping array for AT-iPS cells in ATiPS-262 cells at passage 17, ATiPS-263 cells at passage 27, ATiPS-264 cells at passage 25, and ATiPS-024 cells at passage 25. Compared to parental AT1OS cells, we identified 12 unique structural alterations (Figure 4A, B). Among these genomic alterations, no common chromosomal region was detected in the AT-iPS cells. We also performed exome analysis on the AT-iPS cells to clarify the number of genetic alterations that occur when cells are induced to become pluripotent. The number of bases that our sequencer produced were 18.0, 17.2, 17.4, 17.8 and 18.1 Gb, and mean mapped depths of coverage were 91.7, 89.7, 88.0, 83.3, and 90.0 reads for ATiPS-262, ATiPS-263, ATiPS-264, and ATiPS-024 cells, respectively. In total, 212 SNVs were called. A 23,314-kb copy-neutral loss of heterozygosity (CNLOH) in ATiPS-262 cells, a 3,586-kb deletion in ATiPS-262 cells, and a 234-kb deletion in ATiPS-024 cells involved 2, 37, and 1 SNVs, respectively. These SNVs were removed from the count because the events were caused by large-scale structural mutations rather than single nucleotide substitutions. Furthermore, ambiguously called five SNVs that escaped from the filtering process were manually eliminated (Supplemental Table S2). The numbers of SNVs were 43, 48, 35, and 41 (167 SNVs in total) in ATiPS-262, ATiPS-263, ATiPS-264, and ATiPS-024 cells, respectively (Figure 4B, Supplemental Table S2). Importantly, the identified number of non-synonymous coding bases is larger than that of synonymous coding (Figure 4C). We estimated that 0.48 single nucleotide alterations had occurred per population doubling (PD) in the AT-iPS cells. Single nucleotide change patterns in the 4 AT-iPS cells were summarized in Figure 4D.

### Detection of genomic mutation by the whole exome analysis

The whole exome analysis, in which our samples were compared to the hg19 reference sequence, also detected the homozygous mutation at a splice donor site of the *ATM* gene (chr11:108164206, IVS31 + 2T > A) (Supplemental Table S3). The mutation at the splice donor site is compatible with the truncated *ATM* mRNA that had a deletion of exon 31 (Figure 2A). The detection of this mutation confirms reliability of the whole exome analysis.

### Sensitivity to irradiation in AT-iPS cells

**Figure 5:**
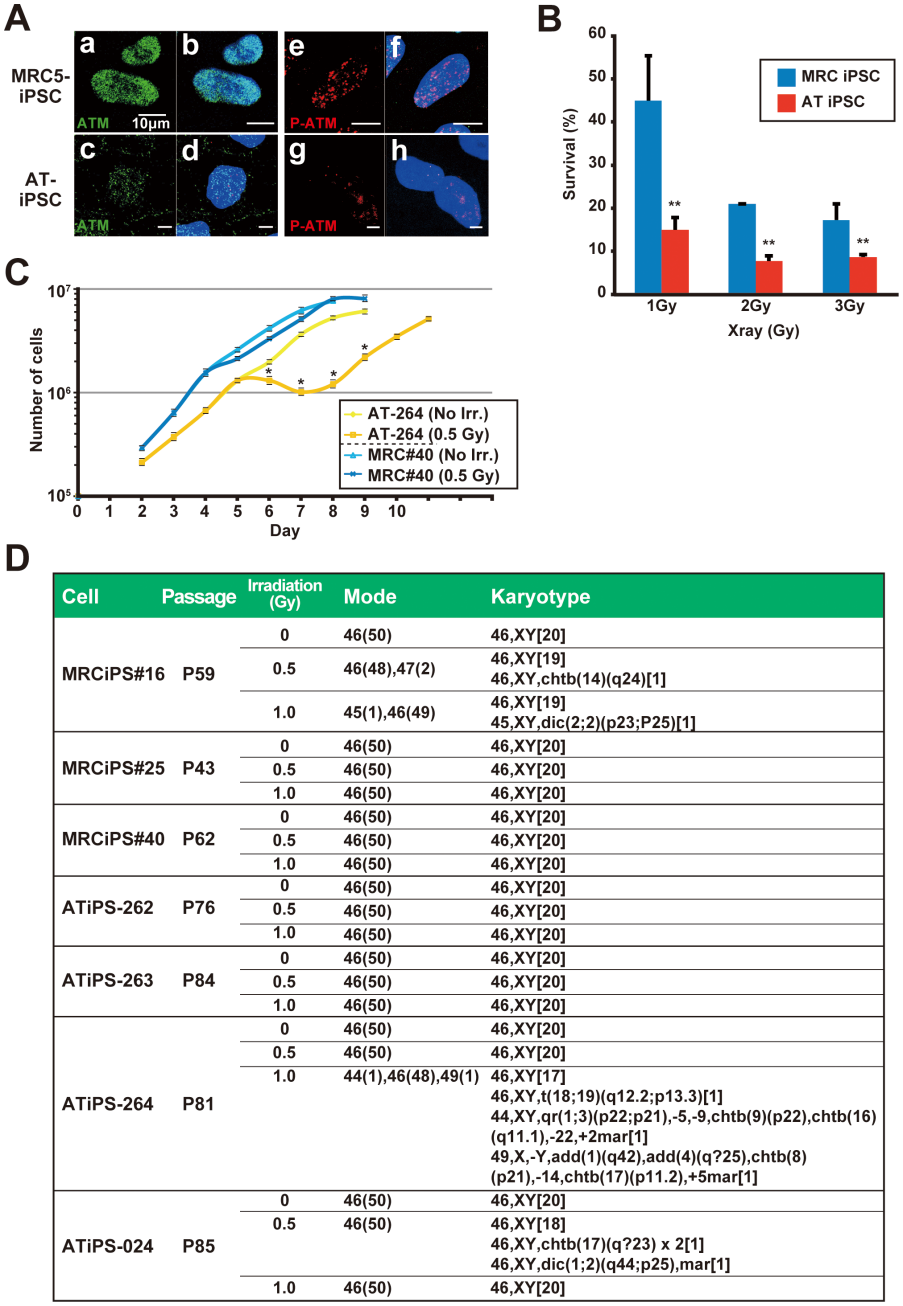
Effect of X-ray irradiation on AT-iPS cells. (A). Immunocytochemistry of ATM (a and c: green) and phosphorylated ATM (e and g: red, labeled as P-ATM). AT-iPS (ATiPS-262) and MRC5-iPS (MRCiPS#16) cells were analyzed at 5 min after 0.5-Gy irradiation. (a), (b), (e) and (f): MRC5-iPS cells; (c), (d), (g) and (h): AT-iPS cells (ATiPS-262). (b) and (d): Merge of ATM and DAPI stain; (f) and (h): Merge of phosphorylated ATM and DAPI stain. (B). Dose effect of irradiation on AT-iPS cells (ATiPS-262, -263, -264, -024) and MRC5-iPS cells (MRCiPS#16, #25, #40). Frequencies of viable cells were calculated from the cell number on 2 days after irradiation at the indicated doses to estimate growth retardation and cell survival. Data for AT-iPS and MRC5-iPS cells were obtained from the quadruplicate and triplicate independent experiments, respectively. Nonirradiated cells were regarded as equal to 100%. Asterisks (**) denote statistically significant with p < 0.01 by student's t-test (1 Gy, p = 1.2 × 10^−3^; 2 Gy, p = 5.1 × 10^−6^; 3 Gy, p = 2.8 × 10^−3^). (C). Effect of irradiation on growth of AT-iPS and MRC5-iPS cells. iPS cells were irradiated at 0.5 Gy on Day 4. Cell number was calculated at the indicated days after cells (10^5^) were seeded on Day 0. Non-irradiated (No Irr.) iPS cells were also shown for control. Asterisks (*) denote statistically significant between irradiated and nonirradiated cells with p < 0.01 by student's t-test (Day 6, p = 3.2 × 10^−4^; Day 7, p = 6.1 × 10^−6^; Day 8, p = 3.8 × 10^−6^; Day 9, p = 9.9 × 10^−7^). (D). Irradiation effect on karyotypes of AT-iPS cells. Mode: 50 cells were analyzed for chromosomal aneuploidy. Number of cells with each chromosomal number is shown in Parentheses. Karyotype: For karyotypic analysis, 20 cells were analyzed and the number of karyotype was shown in brackets.

AT patients and the cells derived from the patients show higher sensitivity to ionizing radiations and to radiomimetic drugs. Thus, we examined the radiosensitivity of AT-iPS cells, and compared it with that of MRC5-iPS cells (Figure 5A). Five minutes after irradiation at 0.5 Gy, the phosphorylation of ATM increased in MRC5-iPS cells. In contrast, ATM protein was undetectable in the non-irradiated state, and was barely induced and phosphorylated in AT-iPS cells even after irradiation.

Two days after X-ray irradiation, cell number was measured in four independent AT-iPS cell clones and three MRC5-iPS cell clones to estimate growth retardation and cell survival (Figure 5B, Supplemental Figure S7A). All AT-iPS clones exhibited markedly lower survival rates than those of MRC5-iPS clones at all different doses examined. At the low dose of irradiation (0.5 Gy), cell growth curve profiles showed that AT-iPS cell growth decreased between day 5 and day 7 post-irradiation, but recovered their proliferation rate at day 8 (Figure 5C). AT-iPS cells exhibited higher sensitivity morphologically to irradiation, compared with MRC5-iPS cells (Supplemental Figure S7B, C). These results indicate that AT-iPS cells have higher radiation sensitivity than the intact iPS cells when growth characteristics are considered.

Karyotypic analyses of AT-iPS cells and MRC5-iPS cells were performed after X-ray irradiation, and did not reveal any significant difference in the radiation sensitivity. Most cells analyzed showed an intact chromosomal pattern even after longer cultivation (ATiPS-264: Passage 81 and 20 months, ATiPS-024: Passage 86 and 22 months). Only low frequencies of chromosomal abnormalities such as chromosomal loss and amplification, deletion, and translocation were detected in AT-iPS cells; MRC5-iPS cells showed similar results (Figure 5D).

### Neural differentiation of iPS cells

**Figure 6:**
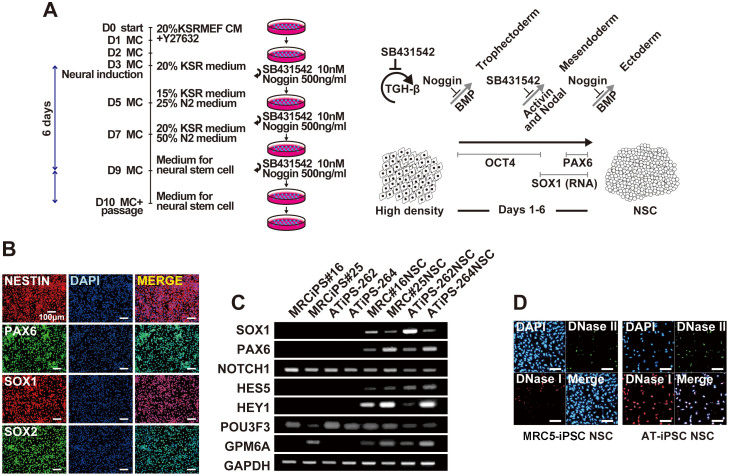
Neural differentiation of AT-iPS cells. (A).Protocol for neural differentiation of AT-iPS cells. Neural differentiation of iPS cells was performed according to the standard protocol^37^. MC: Medium change. (B). Immunocytochemistry of AT-iPS cells (ATiPS-262). (C). RT-PCR analysis on AT-iPS cells (ATiPS-262, ATiPS-264) and MRC5-iPS cells (MRCiPS#16, MRCiPS#25) after neural differentiation. Primers are listed in Supplemental Table S1. (D). Apoptosis of AT-iPS cells after neural differentiation. Apoptosis was detected by the ApopTag ISOL Dual Fluorescence Apoptosis Detection Kit (DNase Types I & II) APT1000 (Millipore). Left panels: neural differentiation of MRC5-iPS cells (MRCiPS#25), right panels: neural differentiation of AT-iPS cells (ATiPS-262).

Since one of the most common symptoms in patients with ataxia telangiectasia is neural impairment, we investigated neural differentiation of AT-iPS cells (ATiPS-262 and -264) and MRC5-iPS cells (MRCiPS#16 and #25) (Figure 6A). AT-iPS cells exhibited neural phenotypes by morphological analysis, immunocytochemistry (Figure 6B), and RT-PCR analysis (Figure 6C, Supplemental Figure S8), and no significant difference between AT-iPS cells and MRC5-iPS cells were detected. However, apoptosis significantly increased after neural differentiation of AT-iPS cells, compared with MRC5-iPS cells (Figure 6D). We obtained consistent results from all iPS cells examined.

## Discussion

Human pluripotent stem cells deficient for the ATM gene have successfully been generated in two ways: Disruption of the ATM gene in human ES cells by genetic manipulation with bacterial artificial chromosome and derivation of disease-specific iPS cells from patients with ataxia telangiectasia^11^,^12^,^13^. The ATM-deficient pluripotent stem cells serve as disease model cells for clarification of pathogenic mechanisms and for screening novel compounds to treat the disease. In this study, we generated iPS cells from fibroblasts (AT1OS) of a human AT patient, and compared them with those from a healthy donor. The AT-iPS cells exhibited the same proliferation activity as wild type-iPS cells (WT-iPS cells), a gene expression profile characteristic of pluripotent stem cells such as ES cells and WT-iPS cells, the capability to differentiate into all three germ layers, and hypersensitivity in growth characteristics to X-ray irradiation. Apoptosis could be induced upon neural differentiation of AT-iPS cells. These results indicate that the established cells kept both characteristics of pluripotent stem cells and *ATM*-deficient cells.

Though normal ATM function was not a prerequisite for the establishment and maintenance of iPS cells, the reprogramming efficiency of the fibroblasts derived from an AT patient was extremely low, suggesting indirect roles of ATM in the somatic reprogramming process. One of the major targets of ATM is p53^14^, and ATM-dependent phosphorylation is directly responsible for p53 activation. ATM and p53 are two proteins that are believed to play a major role in maintaining the integrity of the genome. In spite of having the related function of maintaining the integrity of the genome, p53 is known to serve as a barrier in iPS cell generation. Genetic ablation or decreased amounts of p53 remarkably increases reprogramming efficiency in mouse and human somatic cells^15^,^16^,^17^,^18^,^19^. Thus, ATM and p53 appear to have opposite roles on the reprogramming of differentiated cells to pluripotent cells. ATM kinase phosphorylates a broad range of substrates, and is involved in a wider range of safeguard systems than p53, such as DNA repair, apoptosis, G1/S, intra-S checkpoint and G2/M checkpoints. Thus, p53-independent reprogramming processes may have a crucial need for some ATM functions, and other phosphatidyl-inositol 3-kinase like enzymes, such as ATR, may partially compensate the ATM-deficiency.

Alternatively, telomere damage may explain the low reprogramming efficiency found in AT-derived fibroblasts. Telomeres found at the ends of eukaryotic chromosomes prevent their erosion, facilitate the recruitment of telomere-binding factors and stop the activation of the DNA damage response pathways. In humans, *ATM* deficiency results in accelerated telomere loss, and T lymphocytes derived from AT-patients exhibit frequent telomeric fusions^20^. Mouse cells with short telomeres cannot be reprogrammed to iPS cells despite their normal proliferation rates, probably reflecting the existence of ‘reprogramming barriers’ that abort the reprogramming of cells with uncapped telomeres^21^,^22^.

Unexpectedly, we found that AT-iPS cells did not show any chromosomal instability *in vitro*, i.e., maintenance of intact chromosomes was observed after 80 passages (560 days). Even after X-ray irradiation at low dose, the most of AT-iPS cells still maintained an intact karyotype. In contrast, the parental fibroblastic cell line, AT1OS, showed frequent chromosomal abnormalities, such as deletion, addition and translocation. However, the AT-iPS cells still exhibited hypersensitivity to X-ray irradiation in the growth profile, which are major characteristics of ATM-deficient cells. These results indicate that AT-iPS cells maintain the defective response to ionizing irradiation, but that the defects do not affect maintenance of intact chromosomes.

What are the causes of the differences in the chromosomal stability between AT-iPS cells and AT1OS cells, the source of AT-iPS cells? The major difference between iPS cells and somatic cells may be the ability to undergo unlimited self-renewal. Somatic cells usually have limited growth potential, gradually decline with advancing age, and finally fall into senescence. In contrast, pluripotent stem cells are characterized by unlimited self-renewal and the ability to generate differentiated functional cell types. One of the causes of immortality is the presence of a terminal DNA polymerase capable of synthesizing telomeres, and somatic cell mortality is the result of a progressive loss of the telomeric DNA because of the absence of the immortalizing polymerase. The function of telomerase in terminal DNA elongation is necessary in order to overcome the “end-replication problem” whereby conventional DNA polymerases cannot fully replicate linear DNAs^23^. Telomere erosion (by 50–100 bp per cellular division) limits the replicative capacity of the majority of somatic cells, which do not express active telomerase^24^. In humans, *ATM* deficiency results in accelerated telomere loss in somatic cells, and T lymphocytes derived from AT patients exhibit frequent chromosomal instability^20^.

Response to oxidative stress may be one of the causes of the accelerated telomere loss. It has been suggested that somatic cells, such as fibroblasts and neuronal cells from AT patients are in a chronic state of oxidative stress, which could contribute to their enhanced telomere shortening^25^. ATM protein is suggested to have a role in the prevention or repair of oxidative damage to telomeric DNA, and enhanced sensitivity of telomeric DNA to oxidative damage in AT cells results in accelerated telomere shortening and chromosomal instability. Further study using telomerase inhibitors and anti-oxidants using the human AT-iPS cells may clarify the cause of the difference between somatic cells and iPS cells derived from AT patients.

The number of single-nucleotide mutations per cell genome was estimated from 22 human iPS cells by extensive exome analysis on protein-coding sequences^26^. The exome analysis on the AT-iPS cell lines from 17 to 27 passages after the establishment in this study included not only coding but also untranslated, non-coding RNA, and their adjacent regions, covering up to 93.9 Mb. The number of observed coding mutations during reprogramming was comparable or smaller in all the three lines than those reported by the preceding study^26^, supporting a genetic stability of the AT-iPS cells.

AT is characterized by early onset progressive cerebellar ataxia, oculocutaneous telangiectasia, susceptibility to bronchopulmonary disease, and lymphoid tumors. The pathologic tissues are generally not easily accessible, resulting in a substantial disadvantage for medical and biological studies of the pathogenesis of the disease and for development of novel therapeutic interventions. Generation of *Atm*-deficient mice partially overcomes such difficulties. However, oculocutaneous telangiectasias and histological evidence of neuronal degeneration, which are characteristics of human AT patients, have not seen in these mice, suggesting that the mouse model for AT is limited. Thus, the established human AT-iPS cells described in this study show promise as a tool for clarifying the pathogenesis of AT, and may facilitate development of drugs that inhibit ataxia and hypersensitivity to ionizing radiation.

## Methods

### Ethical statement

Human cells in this study were performed in full compliance with the Ethical Guidelines for Clinical Studies (2008 Notification number 415 of the Ministry of Health, Labor, and Welfare). The cells were banked after approval of the Institutional Review Board at the National Institute of Biomedical Innovation (May 9, 2006).

### Human cells

AT1OS cells were obtained from a ten-year-old Japanese boy (JCRB Cell Bank, Osaka, Japan). The patient history is contained in the original report^27^. The patient was referred to the hospital because of progressive cerebellar ataxia and repeated upper respiratory infection. He raised his head well at 5 months and walked alone at 14 months of age. At the age of 2 years, his parents first noticed his tottering gait. He suffered from severe suppurative tympanitis at 4 years of age, since then he was recurrently afflicted with upper respiratory infections. Before school age, he had already developed a progressive ataxic gait. At the age of 10 years, he could walk alone only a short distance. The neurological examination revealed hyporeflexia, choreoathetosis, oculomotor apraxia and cerebellar dysarthria. Telangiectasia was seen in his bulbar conjunctivae. He showed mild mental retardation (IQ, 72). X-ray computed tomography revealed the fourth ventricular enlargement, suggesting mild cerebellar atrophy. Laboratory tests disclosed a decreased serum level of IgA (17 mg/dl) and a markedly elevated level of α-fetoprotein (560 ng/ml). Serum IgE and IgM were within normal levels. His parents are first cousins.

AT1OS cells were cultured in culture dishes (100 mm, Becton Dickinson). All cultures were maintained at 37°C in a humidified atmosphere containing 95% air and 5% CO_2_. When the cultures reached subconfluence, the cells were harvested with a Trypsin-EDTA solution (cat# 23315, IBL CO., Ltd, Gunma, Japan), and re-plated at a density of 5 × 10^5^ cells in a 100-mm dish. Medium changes were carried out twice a week thereafter. MRC5-iPS cells were maintained on irradiated MEFs as previously described^28^,^29^. MRC5iPS#16 (Fetch), MRC5iPS#25 (Tic), and MRC5iPS#40 (Skipper) were used as controls for AT-iPS cells. MRC5 (ATCC, CCL-171), a parental cell of MRC5-iPS cells, is from lung fibroblasts of 14-week fetus (Caucasian male).

### Generation of iPS cells

AT-iPS cells were generated according to the method as previously described^28^. Briefly, to produce VSV-G (vesicular stomatitis virus G glycoprotein) retroviruses, 293FT cells (Invitrogen) were plated at 2 × 10^6^ cells per 10-cm culture dish with DMEM supplemented with 10% FBS, and incubated overnight. On the next day, the cells were co-transfected with pMXs-OCT4, SOX2, KLF4 or c-MYC, pCL-GagPol, and pHCMV-VSV-G vectors using the TransIT-293 reagent (Mirus Bio LLC, Madison WI). The virus-containing supernatants were collected 48 h after incubation. The supernatants were filtered through a 0.45 μm pore-size filter, centrifuged, and then resuspended in DMEM supplemented with 4 μg/ml polybrene (Nacalai Tesque, Kyoto, Japan). Human AT1OS cells were seeded at 1.0 × 10^5^ cells per well of 6-well plate 24 h before infection. A 1:1:1:1 mixture of OCT3/4, SOX2, KLF4, and c-MYC viruses was added to AT1OS cells^28^,^29^,^30^,^31^. The retrovirus carrying the EGFP gene was infected to estimate infection efficiency in a separate experiment. One-half of the medium was changed every day and colonies were picked up at around day 28.

### RT-PCR

Total RNA was isolated from cells using the TRIzol (Invitrogen) or the RNeasy Plus Mini Kit (Qiagen). cDNA was synthesized from 1 μg of total RNA using Superscript III reverse transcriptase (Invitrogen) with random hexamers according to the manufacturer's instructions. Template cDNA was PCR-amplified with gene-specific primer sets (Supplemental Table S1).

### Quantitative RT-PCR

RNA was extracted from cells using the RNeasy Plus Mini kit (Qiagen). An aliquot of total RNA was reverse transcribed using an oligo (dT) primer. For the thermal cycle reactions, the cDNA template was amplified (ABI PRISM 7900HT Sequence Detection System) with gene-specific primer sets using the Platinum Quantitative PCR SuperMix-UDG with ROX (11743-100, Invitrogen) under the following reaction conditions: 40 cycles of PCR (95°C for 15 s and 60°C for 1 min) after an initial denaturation (95°C for 2 min). Fluorescence was monitored during every PCR cycle at the annealing step. The authenticity and size of the PCR products were confirmed using a melting curve analysis (using software provided by Applied Biosystems) and a gel analysis. mRNA levels were normalized using GAPDH as a housekeeping gene.

### Western blot analysis

Western blot analysis of total cell lysate for p53 and phospho-p53(Ser15) and of nuclear fractions for ATM was performed as described^32^. The membrane filter was probed with the antibodies to p53 (Enzo Life Sci., BML-SA293), phosphor-p53 (S15) (Cell signaling, #9284), and ATM (MBL, PM026), and then incubated with HRP-conjugated antibody to rabbit IgG. The protein signals were detected by ECL detection (Amersham).

### Immunocytochemical analysis

Cells were fixed with 4% paraformaldehyde in PBS for 10 min at 4°C. After washing with PBS and treatment with 0.2% trypsin in PBS (PBST) for 10 min at 4°C, cells were pre-incubated with blocking buffer (10% goat serum in PBS) for 30 min at room temperature, and then reacted with primary antibodies in blocking buffer for 12 h at 4°C. Followed by washing with 0.2% PBST, cells were incubated with secondary antibodies; anti-rabbit or anti-mouse IgG conjugated with Alexa 488 or 546 (1:300) (Invitrogen) in blocking buffer for 1 h at room temperature. Then, the cells were counterstained with DAPI and mounted.

### Karyotypic analysis

Karyotypic analysis was contracted out at Nihon Gene Reserch Laboratories Inc. (Sendai, Japan). Metaphase spreads were prepared from cells treated with 100 ng/mL of Colcemid (Karyo Max, Gibco Co. BRL) for 6 h. The cells were fixed with methanol:glacial acetic acid (2:5) three times, and dropped onto glass slides (Nihon Gene Reserch Laboratories Inc.). Chromosome spreads were Giemsa banded and photographed. A minimum of 10 metaphase spreads were analyzed for each sample, and karyotyped using a chromosome imaging analyzer system (Applied Spectral Imaging, Carlsbad, CA).

### Quantitative fluorescence in situ hybridization (Q-FISH)

We measured telomere length by Q-FISH analysis as previously described^33^,^34^,^35^. The parental cells and iPS cells were subjected to telomere measurements by the telomere fluorescent intensities of the p- and q-arms of all the chromosomes in the metaphase spread individually. The telomere lengths were determined by the median telomere fluorescent unit values.

### Exome sequencing

Approximately 2.0 μg of genomic DNA from each cell sample was sonicated to give a fragment size of 200 bp on a Covaris S220 instrument. After 5–6 cycles of PCR amplification, capture and library preparation were performed with Agilent SureSelect Human All Exon V4 + UTRs + lincRNA (80 Mb), followed by washing, elution, and additional 10-cycle PCR. Enriched libraries were sequenced on an Illumina HiSeq 1000 operated in 101-bp paired-end mode. Image analyses and base calling on all lanes of data were performed using CASAVA 1.8.2 with default parameters.

### Read mapping and variant analysis

Reads from each sample were first trimmed by removing adapters and low quality bases at ends using Trimmomatic 0.22 and then aligned to the hs37d5 sequence (hg19 and decoy sequences) using the Burrows-Wheeler Aligner 0.6.2. Uniquely mapped reads were selected by a custom script, converted from sam to bam using SAMtools 0.1.18, and processed by Picard 1.83 to mark PCR duplicates. Genome Analysis Toolkit (GATK) 2.3–9 was then used to remove the duplicates, perform local realignment and map quality score recalibration to produce calibrated bam files for each sample. Multi-sample callings for SNVs were made by GATK. Target regions for variant detection were expanded to 93.9 Mb in total by following the manufacturer's instruction. The annotated VCF files were then filtered using GATK with a stringent filter setting and custom scripts. Variant calls which failed to pass the following filters were eliminated: QUAL < 400 || QD < 2.0 || MQ < 40.0 || FS > 60.0 || HaplotypeScore > 13.0 || GQ < = 60. When genotype is 0/1, 0/2, or 1/2, only SNVs that meet the following conditions were selected: both of the allelic depths > = 8 && difference of the allelic depths within twofold. When genotype is 0/0, 1/1, or 2/2, only SNVs that meet the following conditions were selected: difference of the allelic depths no less than 32-fold, one allelic depth is 1 and the other is no less than 16, or one allelic depth is 0 and the other is no less than 8. Annotations of altered bases were made using SnpEff 3.1 based on GRCh37.69^36^. Custom Perl scripts and C programs are available at http://github.com/glires/genomics/.

### Structural mutation analysis

The structural mutation analysis by genome-wide SNP genotyping was performed using Illumina HumanCytoSNP-12 v2.1 DNA Analysis BeadChip Kit. The microarray contains approximately 300,000 SNP markers with an average call frequency of > 99%. Subsequent computational and manual analyses were performed using the Illumina KaryoStudio software. The data have been submitted to the GEO database under accession number GSE54576.

### Irradiation

Cells were irradiated by X-ray at 0.87 Gy/min, using MBR-1520R-3 (Hitachi, Tokyo, Japan). Immediately after irradiation, cells were returned to the incubator at 37°C in a humidified atmosphere containing 95% air and 5% CO_2_, and incubated until further processing. Cell number was counted with Vi-CELL 1.00. (Beckman Coulter K.K.,Tokyo, Japan).

### Teratoma formation

AT-iPS cells were harvested by accutase treatment, collected into tubes, and centrifuged. The same volume of Basement Membrane Matrix (354234, BD Biosciences) was added to the cell suspension. The cells (>1 × 10^7^) were subcutaneously inoculated into immuno-deficient, non-obese diabetic (NOD)/severe combined immunodeficiency (SCID) mice (CREA, Tokyo, Japan). After 6 to 10 weeks, the resulting tumors were dissected and fixed with PBS containing 4% paraformaldehyde. Paraffin-embedded tissue was sliced and stained with hematoxylin and eosin (HE). The operation protocols were accepted by the Laboratory Animal Care and the Use Committee of the National Research Institute for Child and Health Development, Tokyo.

### Neural differentiation of iPS cells

We employed the standard protocol for neural differentiation of iPS cells^37^,^38^. Apoptosis was detected by the ApopTag ISOL Dual Fluorescence Apoptosis Detection Kit (DNase Types I & II) APT1000 (Millipore), according to the manufacturer's protocol.

## Author Contributions

A.U. designed experiments. Y.F., M.T., K.O., K.Nakamura, K.Nakabayashi, M.Y.I. and K.T. performed experiments. Y.F., M.T., K.O., K.Nakabayashi, M.Y.I., M.N., K.Hata and K.Hanaoka analyzed data. Y.F., M.T., K.O., K.Nakabayashi, S.T., M.Y.I., M.N., K.Hata, AH, and K.Hanaoka contributed reagents, materials and analysis tools. A.M. and A.U. wrote this manuscript.

## Additional Information

**Accession codes** The SNP genotyping by SNP array data was uploaded to the ncbi web site (GSE47498: Increased X-ray sensitivity and sustained chromosomal stability in Ataxia Telangiectasia-derived induced pluripotent stem (AT-iPS) cells, GSM1151202: AT1OS cells, GSM1151203: ATiPS-262 cells at passage 17, GSM1151204: ATiPS-263 cells at passage 27, GSM1151205: ATiPS-264 cells at passage 25, GSM1151206: ATiPS-024 cells at passage 25). The exome data was uploaded to the DDBJ Sequence Read Archive (DRP001084).

## Supplementary Material

Supplementary Information Supplementary Information

## Figures and Tables

**Table 1:** Telomere length measurement for iPS cells

Cell	Metaphase No. of Metaphase Spreads Examined	Mean Values of Median TFI	Sample TFI/Control TFI	Telomere Length (kbp)
AT1OS	4	5187	0.598	4.13
ATiPS-262	8	16495	1.902	13.14
TIG-1	5	8671	1.000	6.91
ATiPS-264	12	17539	2.263	15.64
TIG-1	7	7751	1.000	6.91
ATiPS-024	14	17654	2.393	16.54
TIG-1	10	7378	1.000	6.91

Telomere length of TIG-1 cells at 34 population doublings is 6.91 kbp.

TFI: Telomere Fluorescence Intensity.
