# Supplementary material for: Ataxia telangiectasia derived iPS cells show preserved x-ray sensitivity and decreased chromosomal instability
Source: Sci Rep. 2014 Jun 27;4:5421. doi: 10.1038/srep05421 (PMC4073166; doi:10.1038/srep05421)
Supplement: Supplementary Information [file srep05421-s1.pdf]

Supplementary Information

Ataxia telangiectasia derived iPS cells show preserved x-ray sensitivity and decreased chromosomal instability

Yoshihiro Fukawatase, Masashi Toyoda, Kohji Okamura, Ken-ichi Nakamura, Kazuhiko Nakabayashi, Shuji Takada, Mayu Yamazaki-Inoue, Akira Masuda, Michiyo Nasu, Kenichiro Hata, Kazunori Hanaoka, Kaiyo Takubo, and Akihiro Umezawa

Inventory of Supplementary Information

Supplemental Figure S1. Southern blot analysis using the probes of the trans genes

Supplemental Figure S2. Low-power view of immunocytochemical analysis of AT-iPS cells using antibodies to Nanog (A), Oct3/4 (A), Sox2 (B), SSEA-4 (C), and Tra-1-60 (D)

Supplemental Figure S3. Hierarchical clustering and principal component analysis (PCA)

Supplemental Figure S4. Protein blot analysis of ATM, p53, and phospho-p53

Supplemental Figure S5. Expression of the endogenous genes in MRC5-iPS cells at different passages (continuous cultivation for up to 1 year and 2 months)

Supplemental Figure S6. Characterization of teratomas generated byMRC5-iPS cells after long-term cultivation (more than one year, passage 77)

Supplemental Figure S7. Effect of X-ray irradiation on AT-iPS cells

Supplemental Figure S8. Expression of genes for PAX6, NANOG, and SOX1 in MRC5-iPS cells

Supplemental Table S1. Primer sets for RT-PCR

Supplemental Table S2. List of 167 SNVs accumulated during the ATM iPS cell culture

Supplemental Table S3. Genomic mutation of the ATM gene in AT-iPS cells and parental AT1OS cells

Supplemental Table S4. Primer sets for quantitative RT-PCR

Supplemental Table S5. Antibodies for immunocytochemistry

OCT3/4

1	MRCiPS#16P30	2
2	MRCiPS#16P25	2
3	MRCiPS#16P14	4
4	ATiPS-262P72	1
5	ATiPS-263P75	3
6	ATiPS-264P69	4
7	ATiPS-024P72	3

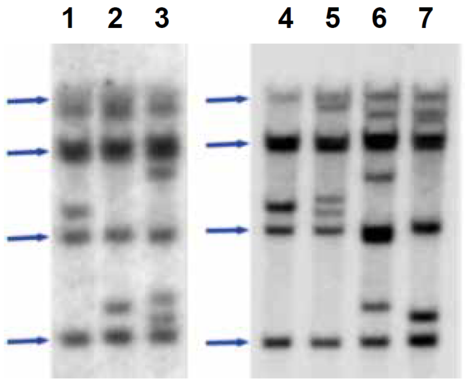

SOX2

1	MRCiPS#16P30	1
2	MRCiPS#16P25	1
3	MRCiPS#16P14	3
4	ATiPS-262P72	3
5	ATiPS-263P75	0
6	ATiPS-264P69	3
7	ATiPS-024P72	6-7

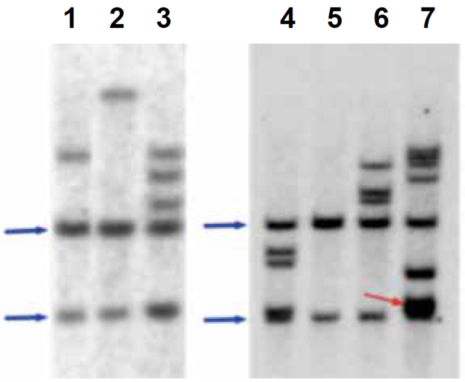

KLF4

1	MRCiPS#16P30	0-1
2	MRCiPS#16P25	2
3	MRCiPS#16P14	3
4	ATiPS-262P72	3
5	ATiPS-263P75	2
6	ATiPS-264P69	2-3
7	ATiPS-024P72	14-15

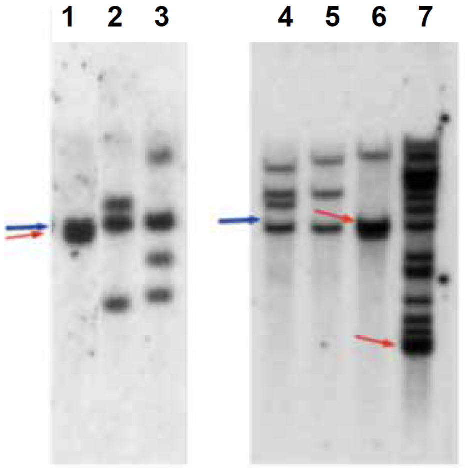

c-MYC

1	MRCiPS#16P30	1
2	MRCiPS#16P25	1
3	MRCiPS#16P14	2
4	ATiPS-262P72	3
5	ATiPS-263P75	1
6	ATiPS-264P69	3
7	ATiPS-024P72	8-9

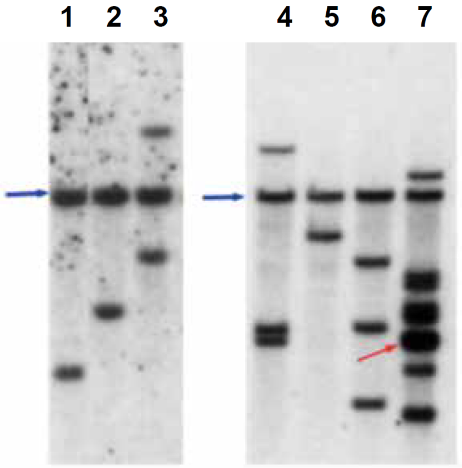

Supplemental Figure S1. Southern blot analysis using the probes of the trans genes
Transgenes were identified by Southern blotting analysis of AT1OS and AT-iPS cells with the probes of OCT4 (383 bp from the start site), SOX2 (424 bp from the stop codon), c-MYC (whole cDNA), and KLF4 (whole cDNA). Copy numbers were analyzed by the bio-imaging analyzer, using copy number controls. The bands corresponding to the exogenous genes were used to calculate copy numbers. The bands indicated by the blue and red arrows correspond to the endogenous gene and the possible exogenous gene, respectively.

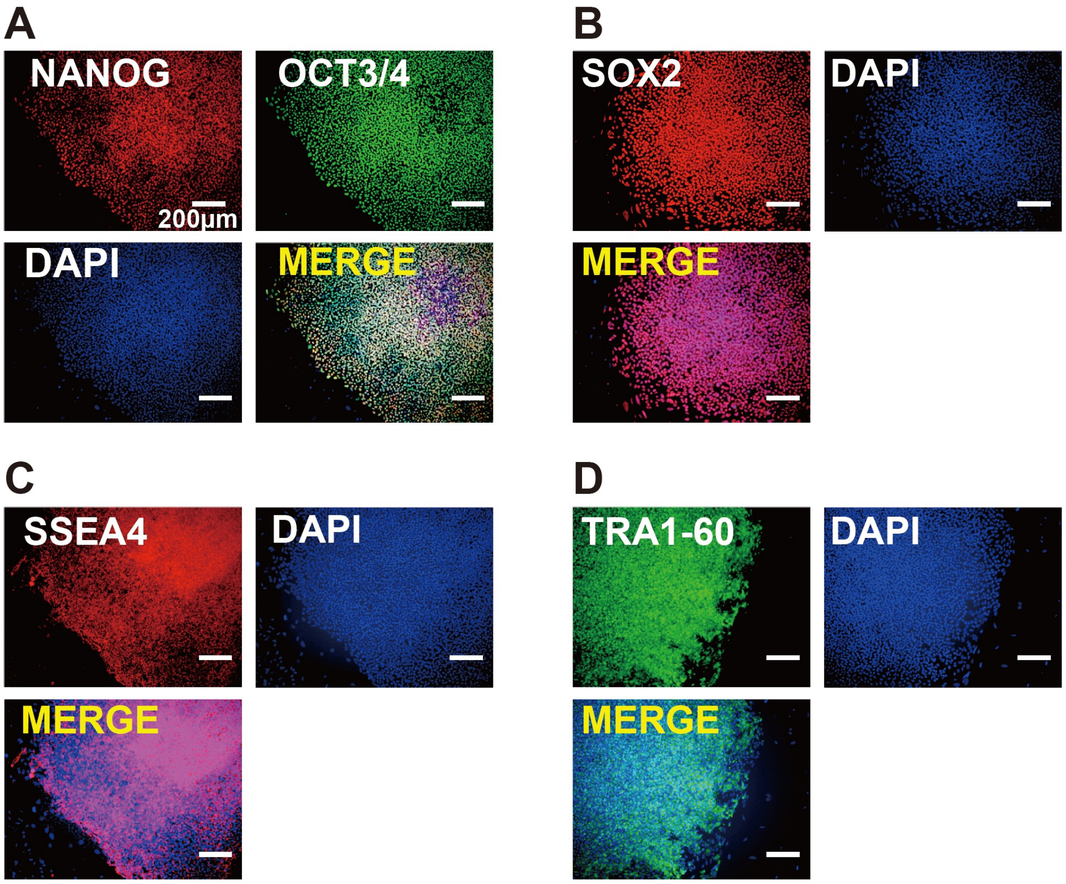

Supplemental Figure S2. Low-power view of immunocytochemical analysis of AT-iPS cells using antibodies to Nanog (A), Oct3/4 (A), Sox2 (B), SSEA-4 (C), and Tra-1-60 (D)

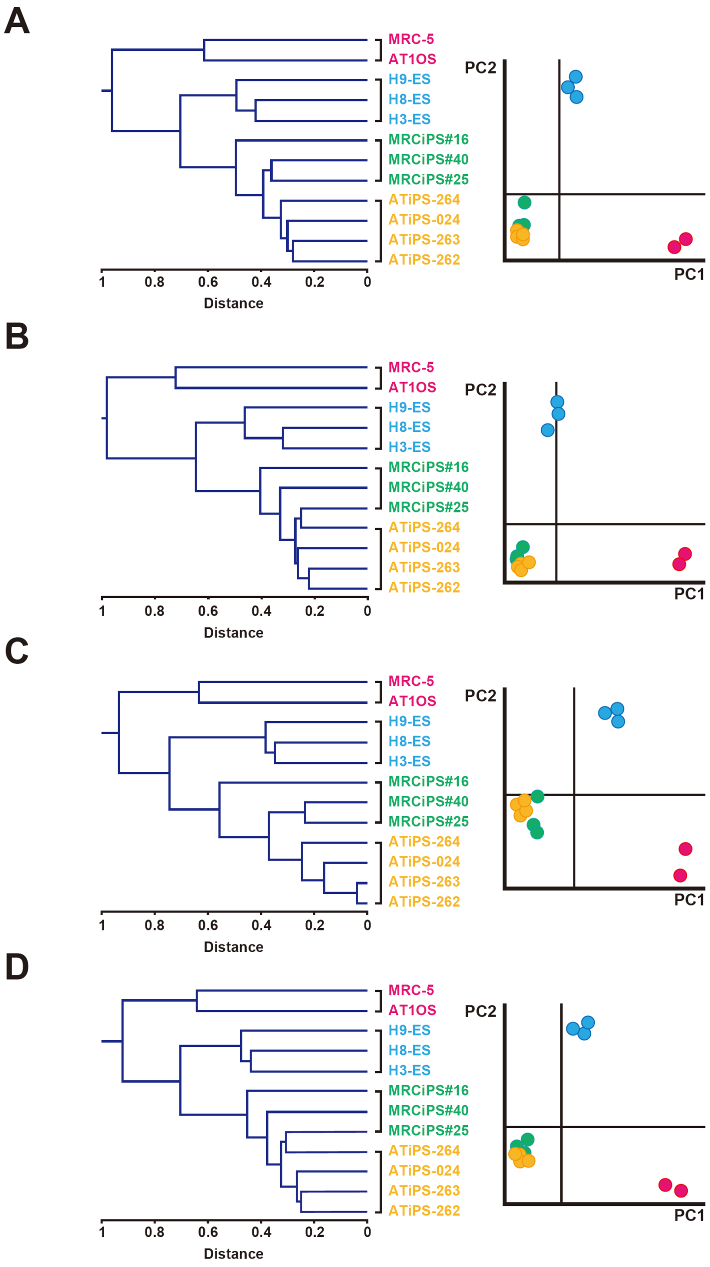

Supplemental Figure S3. Hierarchical clustering and principal component analysis (PCA)
For analysis of gene expression microarray, cDNA was reverse-transcribed from each RNA sample, using Low RNA Input Fluorescent Linear Amplification Kits (Agilent), and cRNA then transcribed and fluorescently labeled with Cy3. cRNA was purified using an Agilent One Color Spike Mix Kit (Agilent Technologies). We hybridized 1,650 ng of Cy3-labeled and amplified cRNA to Agilent 4 x 44 K whole human genome microarrays and processed it according to the manufacturer's instructions. The array was scanned using an Agilent G2505B DNA microarray scanner (Agilent Technologies). The image files were extracted using Feature Extraction software (version 10.7.3.1, Agilent Technologies) background subtraction and dye normalization. The data were analyzed using GeneSpring GX 10.0 (Agilent Technologies). To analyze the expression data of genes (A: all genes, B: neural genes, C: DNA damage-related genes, D: cell cycle-related genes) in an unsupervised manner by gene chip array, we used hierarchical clustering and principal component analysis (Sharov AA, et al., Bioinformatics, 21: 2548, 2005; Saeed AI, et al., Biotechniques, 34: 374, 2003). The hierarchical clustering techniques classify data by similarity and the results are represented by dendrogram. PCA is a multivariate analysis technique which finds major patterns in data variability. Hierarchical clustering and PCA were performed to group iPS cells, parental cells, and ES cells into subcategories.

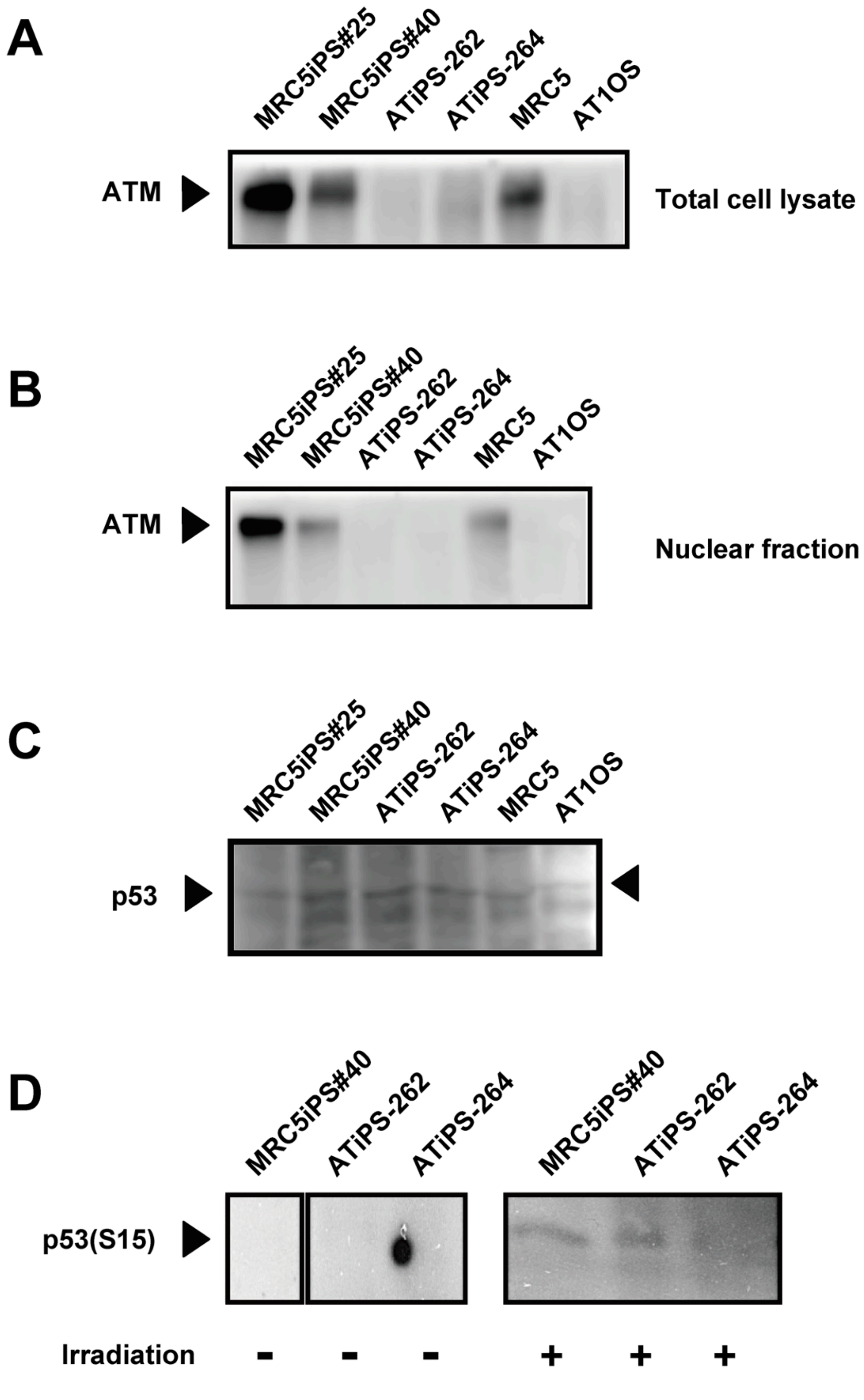

Supplemental Figure S4. Protein blot analysis of ATM, p53, and phospho-p53
(A, B) Expression of ATM in iPS cells at the growth phase. A: Total cell lysate, B: Nuclear fraction. (C) Expression of p53 in iPS cells at the growth phase. (D) Expression of phospho-p53 in iPS cells without irradiation (left panel) and 2 days after 0.5 Gy irradiation (right panel).

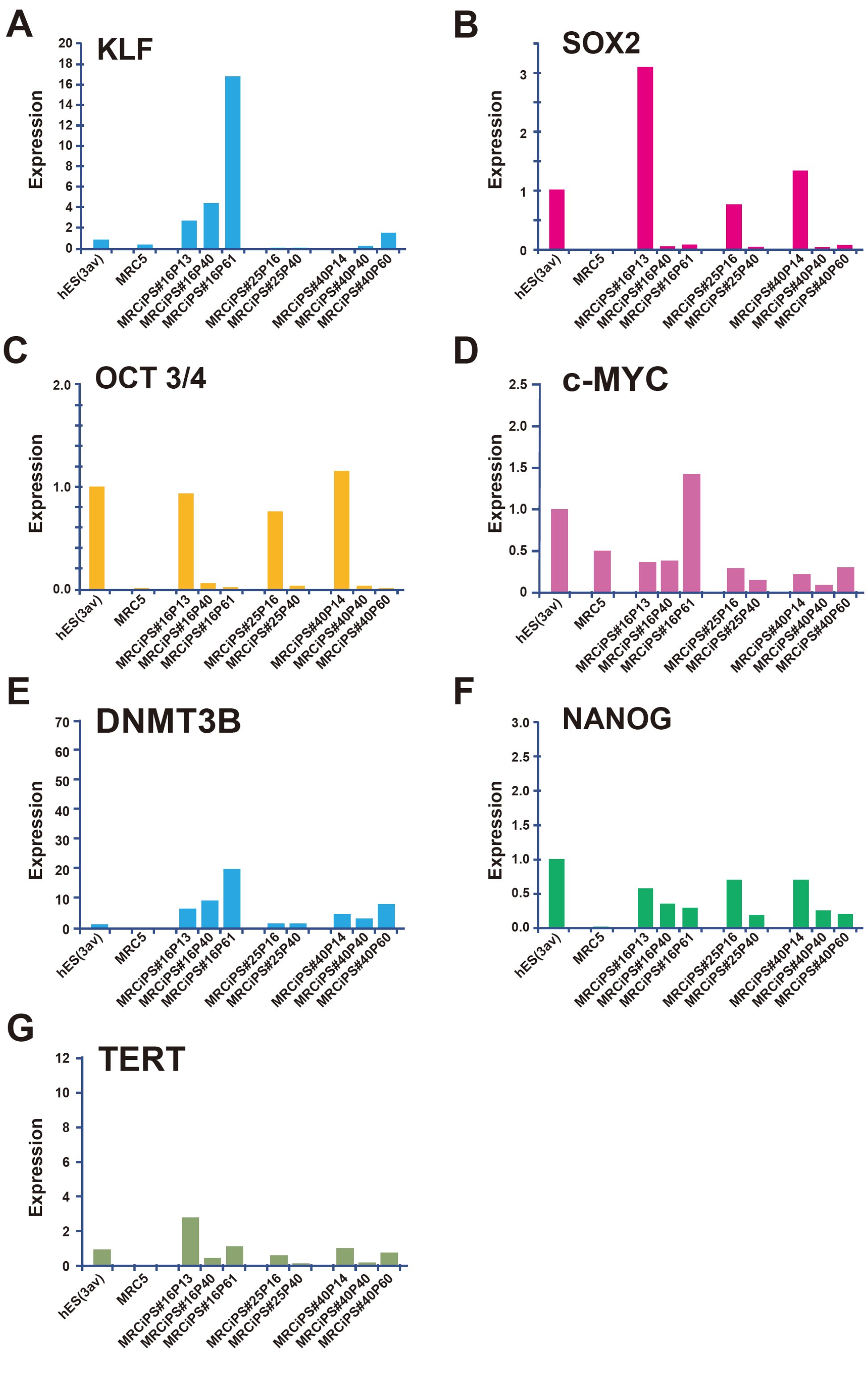

Supplemental Figure S5. Expression of the endogenous genes in MRC5-iPS cells at different passages (continuous cultivation for up to 1 year and 2 months)
(A) Expression of the endogenous *KLF4* gene. (B) Expression of the endogenous *SOX2* gene. (C) Expression of the endogenous *OCT-3/4*gene. (D) Expression of the endogenous *c-MYC* gene. (E) Expression of the *DNMT-3B* gene. (F) Expression of the *NANOG* gene. (G) Expression of the *TERT* gene.

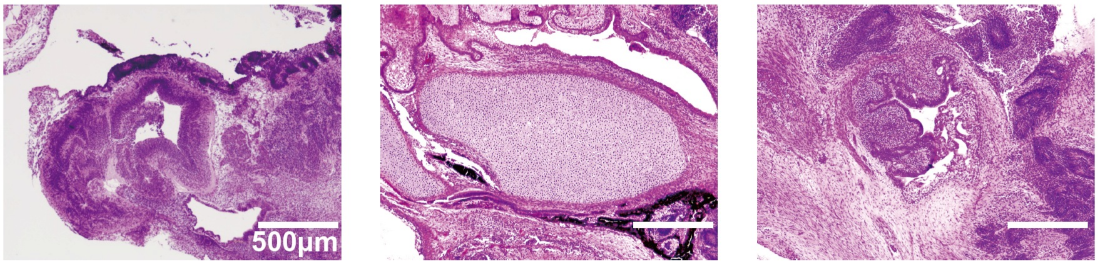

Supplemental Figure S6. Characterization of teratomas generated byMRC5-iPS cells after long-term cultivation (more than one year, passage 77)
Teratoma formation after injection into immunodeficient mice (left panel: neuroectodermal tissue, middle panel: cartilage, right panel: intestine).

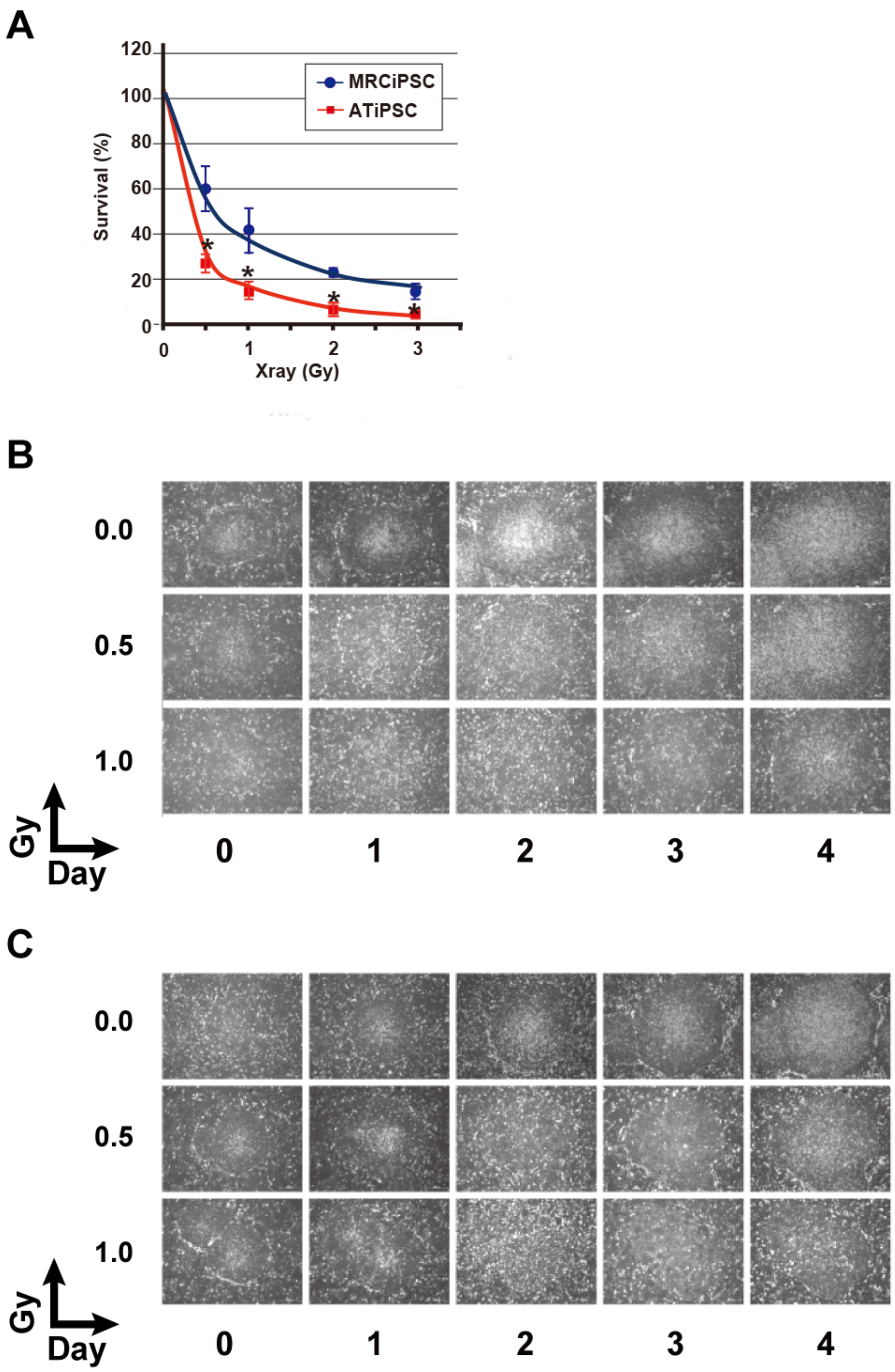

Supplemental Figure S7. Effect of X-ray irradiation on AT-iPS cells
(A) Dose effect of irradiation on AT-iPS and MRC5-iPS cells. Frequencies of viable cells were calculated from the cell number on 2 days after irradiation at the indicated doses to estimate growth retardation and cell survival. Nonirradiated cells were regarded as equal to 100%. Asterisks (*) denote statistically significant with p<0.01 by student's t-test. (B, C) Phase contrast photograph of MRC5-iPS (B) and AT-iPS (C) cells after irradiation To investigate effect of X-ray irradiation on AT-iPS cell morphology, MRC5-iPS (MRCiPS#16) and AT-iPS (ATiPS-262) cells were exposed to 0.5- or 1.0-Gy irradiation on Day 0, and observed for 4 days.

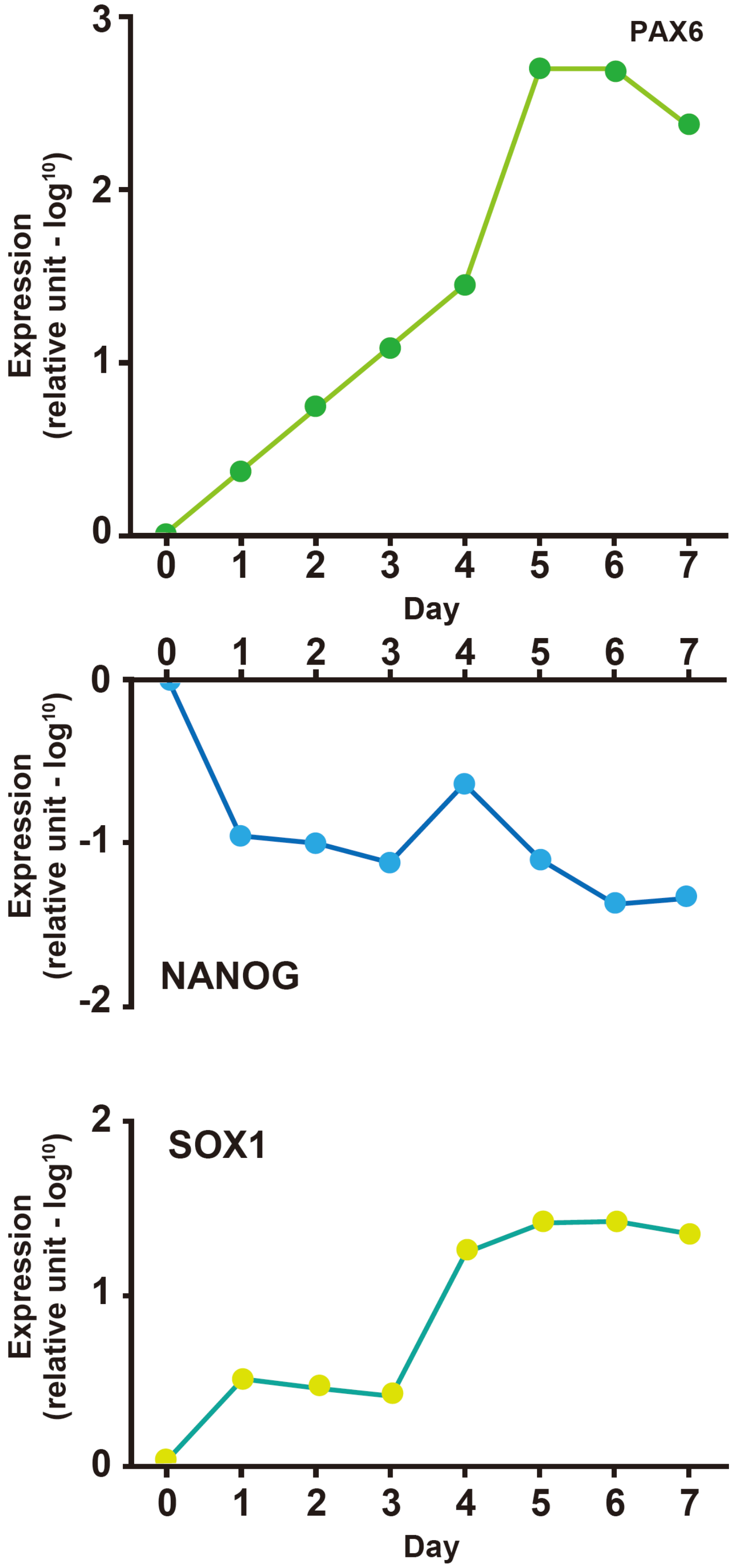

Supplemental Figure S8. Expression of genes for PAX6, NANOG, and SOX1 in MRC5-iPS cells
Gene expression was monitored by quantitative RT-PCR during neural differentiation of MRC5-iPS cells. Expression level without induction (Day 0) was regarded as equal to $\log_{10}1$ (=0).

Supplemental Table S1. Primer sets for RT-PCR

Gene Product	Forward (5'→3')	Reverse (5'→3')
**Pluripotency**		
GAPDH	GCTCAGACACCATGGGGAAGGT	GTGGTGCAGGAGGCATTGCTGA
OCT3/4	AGTAGACGGCATCGCAGCTTG	AGCCAGGTCCGAGGATCAAC
SOX2	AGTAGACGGCATCGCAGCTTG	GCTTCAGCTCCGTCTCCATC
KLF4	GGCACTACCGTAAACACACG	GCTAGCTTGCCAAACCTACAG
c-MYC	ACCGAGGAGAATGTCAAGAG	GCTAGCTTGCCAAACCTACAG
**Ataxia Terangiectasia**		
ATM	AGTGGCTTAGGAGGAGCTTGG	GACCCACTTCTCCCAAGCAGC
**Neural genes**		
SOX1	CAATGCGGGGAGGAGAAGTC	CTCTGGACCAAACTGTGGCG
PAX6	AATAACCTGCCTATGCAACCC	AACTTGAACTGGAACTGACACAC
NOTCH1	ACTGTGAGGACCTGGTGGAC	TTGTAGGTGTTGGGGAGGTC
HES5	GCCCGGGGTTCTATGATATT	GAGTTCGGCCTTCACAAAAG
HEY1	CGAGGTGGAGAAGGAGAGTG	CTGGGTACCAGCCTTCTCAG
POU3F3	GTTCTCGCAGACCACCATCT	CGATAGAGGTCCGCTTCTTG
GPM6A	TGAGATGGCAAGAACTGCTG	CCAGGCCAACATGAAAAGAT

Supplemental Table S2. List of 167 SNVs accumulated during the ATM iPS cell culture								
hg19	Coordinate	Genotype					Appearance line	Annotation by SnpEff
		AT1OS	ATiPS-262	ATiPS-263	ATiPS-264	ATiPS-024		
chr1	17046361	AA	n/a	n/a	n/a	AT	ATiPS-024	INTRON
chr1	23520515	GG	AG	GG	GG	GG	ATiPS-262	SYNONYMOUS_CODING
chr1	32372672	AA	AA	AT	AA	AA	ATiPS-263	DOWNSTREAM
chr1	38175101	GG	GT	GG	GG	GG	ATiPS-262	DOWNSTREAM
chr1	44410518	GG	GG	GG	AG	GG	ATiPS-264	INTRON
chr1	55148429	GG	GG	GG	GG	AG	ATiPS-024	INTRON
chr1	67468901	GG	GG	GT	GG	GG	ATiPS-263	DOWNSTREAM
chr1	70611030	CC	CC	CC	AC	CC	ATiPS-264	DOWNSTREAM
chr1	75862155	TT	AT	TT	TT	TT	ATiPS-262	INTRON
chr1	81106915	GG	CG	GG	GG	GG	ATiPS-262	INTRON
chr1	89730406	AA	AA	AG	AA	AA	ATiPS-263	EXON
chr1	89736947	TT	TT	TT	TT	CT	ATiPS-024	DOWNSTREAM
chr1	103449656	CC	CC	CC	CT	CC	ATiPS-264	INTRON
chr1	150776858	TT	TT	TT	GT	TT	ATiPS-264	DOWNSTREAM
chr1	155220609	GG	GG	GG	GG	AG	ATiPS-024	DOWNSTREAM
chr1	177226186	GG	CG	GG	GG	GG	ATiPS-262	EXON
chr1	179526464	AA	AG	AA	AA	AA	ATiPS-262	DOWNSTREAM
chr1	180080520	GG	GG	CG	GG	GG	ATiPS-263	DOWNSTREAM
chr1	185088801	GG	GG	GG	GG	CG	ATiPS-024	DOWNSTREAM
chr1	197414036	TT	n/a	n/a	CT	n/a	ATiPS-264	DOWNSTREAM
chr1	205627826	GG	GG	AG	GG	GG	ATiPS-263	EXON
chr1	238051925	GG	GT	GG	GG	GG	ATiPS-262	INTRON
chr1	240076732	AA	AA	AG	AA	AA	ATiPS-263	DOWNSTREAM
chr2	11732882	CC	CC	CC	CT	CC	ATiPS-264	DOWNSTREAM
chr2	31491127	CC	CT	CC	CC	CC	ATiPS-262	UTR_3_PRIME
chr2	55561621	AA	AA	AT	AA	AA	ATiPS-263	NON_SYNONYMOUS_CODING
chr2	71901258	CC	CC	CC	AC	CC	ATiPS-264	INTRON
chr2	73993929	TT	TT	TT	CT	TT	ATiPS-264	DOWNSTREAM
chr2	85967516	CC	CC	CC	CT	CC	ATiPS-264	INTERGENIC
chr2	96780986	CT	CC	n/a	n/a	n/a	ATiPS-262	SYNONYMOUS_CODING
chr2	116573324	GG	GG	GG	GG	CG	ATiPS-024	INTRON
chr2	141081476	TT	TT	CT	TT	TT	ATiPS-263	NON_SYNONYMOUS_CODING
chr2	141459901	GG	GG	GT	GG	GG	ATiPS-263	INTRON
chr2	160139477	CC	CC	CC	AC	CC	ATiPS-264	NON_SYNONYMOUS_CODING
chr2	170629917	AA	AA	AG	AA	AA	ATiPS-263	DOWNSTREAM
chr2	183538039	AA	AA	AA	AA	AG	ATiPS-024	INTERGENIC
chr3	54615745	TT	AT	TT	TT	TT	ATiPS-262	INTRON
chr3	102384138	CC	CC	CT	CC	CC	ATiPS-263	INTRON
chr3	102868688	TT	TT	TT	TT	GT	ATiPS-024	INTERGENIC
chr3	109049192	AA	AG	AA	AG	AA	ATiPS-262	DOWNSTREAM
chr3	112080828	AA	AG	AA	AA	AA	ATiPS-262	UTR_3_PRIME
chr3	113432774	CC	CC	CC	CC	CT	ATiPS-024	INTERGENIC
chr3	133467420	GG	AG	GG	GG	GG	ATiPS-262	EXON
chr3	142396838	GG	GG	GT	GG	GG	ATiPS-263	DOWNSTREAM
chr3	168628632	GG	GG	AG	GG	GG	ATiPS-263	INTRON
chr3	171318567	GG	GG	GG	CG	GG	ATiPS-264	DOWNSTREAM
chr3	178947877	AA	AC	AA	AA	AA	ATiPS-262	DOWNSTREAM
chr3	180704796	GG	GT	GG	GG	GG	ATiPS-262	DOWNSTREAM
chr3	193272796	GG	GG	GG	GG	GT	ATiPS-024	DOWNSTREAM
chr3	193826944	AA	AA	AA	AG	AA	ATiPS-264	EXON
chr4	1228787	CC	CC	AC	CC	CC	ATiPS-263	DOWNSTREAM
chr4	2928506	AA	AA	AG	AA	AA	ATiPS-263	DOWNSTREAM
chr4	8322220	TT	TT	CT	TT	TT	ATiPS-263	EXON
chr4	31196087	CC	CC	CC	CT	CC	ATiPS-264	EXON
chr4	73941712	GG	GG	GG	GG	GT	ATiPS-024	DOWNSTREAM
chr4	96087150	GG	AG	GG	GG	GG	ATiPS-262	UTR_3_PRIME
chr4	100818376	TT	CT	CT	TT	CT	ATiPS-262	DOWNSTREAM
chr4	106180889	CC	CC	AC	CC	CC	ATiPS-263	NON_SYNONYMOUS_CODING
chr4	113569490	GG	GG	GG	GG	CG	ATiPS-024	DOWNSTREAM
chr4	123128692	n/a	GG	n/a	n/a	GT	ATiPS-262	INTRON

chr4	153542493	CC	CC	CG	CC	CC	ATiPS-263	UTR_3_PRIME
chr4	156618153	CC	CC	CT	CC	CC	ATiPS-263	EXON
chr4	184687721	GG	GG	GG	GG	GT	ATiPS-024	INTERGENIC
chr4	187177356	CC	AC	CC	CC	CC	ATiPS-262	DOWNSTREAM
chr5	25143028	TT	TT	TT	CT	TT	ATiPS-264	INTRON
chr5	104434330	n/a	GG	AG	GG	GG	ATiPS-263	INTRON
chr5	104434333	n/a	AA	AG	AA	AA	ATiPS-263	INTRON
chr5	158123482	CC	AC	CC	CC	CC	ATiPS-262	DOWNSTREAM
chr5	170240939	GG	GG	GG	GG	AG	ATiPS-024	DOWNSTREAM
chr5	171341302	TT	CT	TT	TT	TT	ATiPS-262	INTRON
chr6	27782841	CC	CG	CC	CC	CC	ATiPS-262	DOWNSTREAM
chr6	30568494	CC	CC	CT	CC	CC	ATiPS-263	DOWNSTREAM
chr6	32627777	CG	n/a	CG	GG	n/a	ATiPS-264	DOWNSTREAM
chr6	39869982	CC	CC	CT	CC	CC	ATiPS-263	DOWNSTREAM
chr6	41349518	GG	GG	GG	GG	AG	ATiPS-024	INTERGENIC
chr6	112197949	GG	GG	GG	GG	GT	ATiPS-024	INTERGENIC
chr6	135376355	GG	GG	GG	GT	GG	ATiPS-264	EXON
chr7	6369570	TT	TT	CT	TT	TT	ATiPS-263	DOWNSTREAM
chr7	40102449	TT	TT	CT	TT	TT	ATiPS-263	EXON
chr7	45231254	AA	AA	AA	AC	AA	ATiPS-264	INTERGENIC
chr7	98286613	CT	CT	n/a	n/a	TT	ATiPS-024	INTERGENIC
chr7	127236023	GG	GG	CG	GG	GG	ATiPS-263	DOWNSTREAM
chr7	140481475	CC	CC	AC	CC	CC	ATiPS-263	NON_SYNONYMOUS_CODING
chr7	149545447	GG	GG	GG	GG	GT	ATiPS-024	DOWNSTREAM
chr7	155570934	n/a	GG	CG	GG	GG	ATiPS-263	UTR_3_PRIME
chr8	22011577	CC	AC	CC	CC	CC	ATiPS-262	INTRON
chr8	39138741	CC	CC	CC	CC	AC	ATiPS-024	DOWNSTREAM
chr8	70585349	GG	GG	GT	GG	GG	ATiPS-263	NON_SYNONYMOUS_CODING
chr8	95938575	CC	CC	CT	CC	CC	ATiPS-263	DOWNSTREAM
chr8	128751266	GG	n/a	GG	n/a	AG	ATiPS-024	DOWNSTREAM
chr8	134537989	AA	AA	AA	AG	AA	ATiPS-262	INTRON
chr8	136659160	TT	GT	TT	TT	TT	ATiPS-262	INTRON
chr8	139142934	TT	AT	TT	TT	TT	ATiPS-262	DOWNSTREAM
chr8	143310818	TT	AT	TT	TT	TT	ATiPS-262	INTRON
chr9	5308221	CC	CC	CC	CC	CT	ATiPS-024	INTERGENIC
chr9	20346391	AC	n/a	AA	AA	AC	ATiPS-264	DOWNSTREAM
chr9	20360870	AA	AA	AA	AA	AG	ATiPS-024	DOWNSTREAM
chr9	79325180	CC	CC	CT	CC	CC	ATiPS-263	SYNONYMOUS_CODING
chr9	95218571	GG	GG	GG	GG	GT	ATiPS-024	INTRON
chr9	120786585	GG	GG	GG	GT	GG	ATiPS-264	INTERGENIC
chr9	126028641	GG	GG	GG	GG	CG	ATiPS-024	INTRON
chr9	126101295	GG	GT	GG	GG	GG	ATiPS-262	INTERGENIC
chr9	128083622	AA	AT	AA	AA	AA	ATiPS-262	INTRON
chr9	129975143	CC	CC	CC	CG	CC	ATiPS-264	DOWNSTREAM
chr9	131303522	AG	AG	AG	AG	GG	ATiPS-024	DOWNSTREAM
chr10	5019882	CC	CC	CC	CC	AC	ATiPS-024	INTRON
chr10	5804490	n/a	n/a	n/a	AT	AA	ATiPS-024	DOWNSTREAM
chr10	5956156	AA	AG	AA	AA	AA	ATiPS-262	DOWNSTREAM
chr10	7514066	AA	AA	AG	AA	AA	ATiPS-263	INTERGENIC
chr10	22649763	AA	AA	AA	AA	AG	ATiPS-024	INTRON
chr10	69994936	AA	AA	AC	AA	AA	ATiPS-263	INTRON
chr10	70644958	AA	AA	AT	AA	AA	ATiPS-263	INTRON
chr10	90588318	TT	TT	TT	TT	CT	ATiPS-024	NON_SYNONYMOUS_CODING
chr10	103503641	CC	CC	CC	CC	CT	ATiPS-024	INTERGENIC
chr11	10022585	AT	AT	AT	AA	AT	ATiPS-024	INTRON
chr11	10024311	AG	AG	AG	AA	AG	ATiPS-264	INTRON
chr11	67821556	TT	TT	TT	TT	GT	ATiPS-024	DOWNSTREAM
chr11	121454351	GG	GG	GG	GG	GT	ATiPS-024	INTRON
chr11	125775210	GG	GG	GT	GG	GG	ATiPS-263	DOWNSTREAM
chr12	7019140	CC	CC	CC	CC	CT	ATiPS-024	DOWNSTREAM
chr12	73046250	GG	CG	n/a	GG	n/a	ATiPS-262	NON_SYNONYMOUS_CODING
chr12	81205190	GG	AG	GG	GG	GG	ATiPS-262	INTRON
chr12	100930754	AA	AT	AA	AA	AA	ATiPS-262	NON_SYNONYMOUS_CODING

chr12	126451607	AA	AA	AA	AG	AA	ATiPS-264	EXON
chr12	126471592	TT	n/a	GT	n/a	n/a	ATiPS-263	DOWNSTREAM
chr13	37679268	n/a	GT	GG	GG	GG	ATiPS-262	NON_SYNONYMOUS_CODING
chr13	47844174	GG	GG	GG	GG	AG	ATiPS-024	INTERGENIC
chr14	22294141	GG	GT	GG	GG	GG	ATiPS-262	EXON
chr14	24776029	CC	CC	CC	CC	CG	ATiPS-024	DOWNSTREAM
chr14	36605081	n/a	GG	n/a	AG	GG	ATiPS-264	DOWNSTREAM
chr14	39949428	AA	AA	AG	AA	AA	ATiPS-263	INTRON
chr14	54878343	GG	GG	AG	GG	GG	ATiPS-263	EXON
chr14	78390727	GG	GG	GT	GG	GG	ATiPS-263	EXON
chr14	94173575	CC	CC	CC	CG	CC	ATiPS-264	DOWNSTREAM
chr15	25367604	AA	AA	AT	AA	AA	ATiPS-263	EXON
chr15	26379662	TT	TT	TT	CT	TT	ATiPS-264	DOWNSTREAM
chr15	51697408	CC	CC	CC	CC	AC	ATiPS-024	DOWNSTREAM
chr16	20348036	n/a	GG	GG	AG	GG	ATiPS-264	EXON
chr16	48220887	TT	TT	TT	TT	CT	ATiPS-024	DOWNSTREAM
chr16	70902604	TT	TT	TT	GT	TT	ATiPS-264	DOWNSTREAM
chr16	74926294	GG	GG	GG	GG	AG	ATiPS-024	DOWNSTREAM
chr16	89212249	CC	CT	CC	CC	CC	ATiPS-262	DOWNSTREAM
chr17	7187283	CC	CC	CT	CC	CC	ATiPS-263	DOWNSTREAM
chr17	34251763	GG	GG	GT	GG	GG	ATiPS-263	INTRON
chr17	37665793	TT	TT	TT	CT	TT	ATiPS-264	INTRON
chr17	39595665	GG	GG	GG	AG	GG	ATiPS-264	INTRON
chr17	67186388	TT	AT	TT	TT	TT	ATiPS-262	INTRON
chr18	32833236	CC	AC	CC	CC	CC	ATiPS-262	EXON
chr18	50589685	GG	GG	GG	GG	GT	ATiPS-024	EXON
chr19	7509353	GG	GG	GG	AG	GG	ATiPS-264	INTRON
chr19	32530396	n/a	AT	n/a	AA	AT	ATiPS-264	INTRON
chr19	35941064	TT	TT	AT	TT	TT	ATiPS-263	NON_SYNONYMOUS_CODING
chr19	38655416	CC	CC	CT	CC	CC	ATiPS-263	NON_SYNONYMOUS_CODING
chr19	48305688	GG	n/a	AG	GG	GG	ATiPS-263	NON_SYNONYMOUS_CODING
chr19	50758526	GG	GG	GG	AG	GG	ATiPS-264	INTRON
chr20	16360019	TT	CT	TT	TT	TT	ATiPS-262	SYNONYMOUS_CODING
chr20	16360348	CC	CT	CC	CC	CC	ATiPS-262	NON_SYNONYMOUS_CODING
chr20	25225988	AA	AG	AA	AA	AA	ATiPS-262	INTERGENIC
chr20	31897619	GG	GG	GG	GT	GG	ATiPS-264	DOWNSTREAM
chr20	35090292	CC	CG	CC	CC	CC	ATiPS-263	INTRON
chr21	19617501	CC	CC	n/a	CT	CC	ATiPS-264	INTRON
chr21	30715165	AA	AA	AA	AC	AA	ATiPS-264	INTRON
chr22	22843758	CC	CG	CC	CC	CC	ATiPS-262	UTR_5_PRIME
chr22	23482590	GG	AG	GG	GG	GG	ATiPS-262	DOWNSTREAM
chr22	32547086	CT	CT	n/a	n/a	CC	ATiPS-024	INTRON
chrX	100245471	CC	CC	AC	CC	CC	ATiPS-263	INTRON
chrX	134169749	AA	AA	TT	AA	AA	ATiPS-263	DOWNSTREAM

Supplemental Table S3, related to Figure 4. Genomic mutation of the ATM gene in AT-iPS cells and parental AT1OS cells

hp19	Position	Ref	Change	Type	Genotype											Gene	Effect
					C1	C2	C3	C4	C5	C6	AT1OS	262	263	264	024		
11	108093208	A	T	SNV	A/T	ref	A/T	T	ref	T	ref	ref	ref	ref	ref	ATM	UPSTREAM: 1048 bases
11	108098460	*	-AA	DEL	ref	ref	ref	T	ref	T	ref	ref	ref	ref	ref	ATM	INTRON
11	108121426	A	T	SNV	ref	ref	A/T	A/T	ref	ref	ref	ref	ref	ref	ref	ATM	INTRON
11	108126944	T	C	SNV	ref	ref	ref	ref	ref	T/C	ref	ref	ref	ref	ref	ATM	SYNONYMOUS_CODING
11	108129657	A	G	SNV	ref	ref	ref	G	ref	G	ref	ref	ref	ref	ref	ATM	INTRON
11	108129794	A	G	SNV	ref	ref	ref	ref	A/G	ref	ref	ref	ref	ref	ref	ATM	NON_SYNONYMOUS_CODING
11	108159732	C	T	SNV	C/T	ref	C/T	ref	ref	ref	ref	ref	ref	ref	ref	ATM	NON_SYNONYMOUS_CODING
11	108164206	T	A	SNV	ref	ref	ref	ref	ref	ref	A	A	A	A	A	ATM	TRANSCRIPT: ENST00000531957
11	108164206	T	A	SNV	ref	ref	ref	ref	ref	ref	A	A	A	A	A	ATM	SPLICE_SITE_DONOR
11	108166232	T	G	SNV	T/G	ref	T/G	ref	ref	ref	ref	ref	ref	ref	ref	ATM	UTR_3_PRIME: 554 bases from CDS
11	108183167	A	G	SNV	G	G	G	G	G	G	G	G	G	G	G	ATM	TRANSCRIPT: ENST00000533690
11	108188204	A	G	SNV	ref	ref	ref	A/G	ref	ref	ref	ref	ref	ref	ref	ATM	TRANSCRIPT: ENST00000533690
11	108206666	A	T	SNV	ref	ref	A/T	ref	ref	ref	ref	ref	ref	ref	ref	ATM	TRANSCRIPT: ENST00000525056
11	108225661	A	G	SNV	A/G	ref	A/G	G	ref	G	ref	ref	ref	ref	ref	ATM	TRANSCRIPT: ENST00000527181
11	108236750	C	T	SNV	ref	ref	ref	ref	ref	ref	T	T	T	T	T	ATM	TRANSCRIPT: ENST00000524792
11	108236783	G	T	SNV	G/T	ref	G/T	T	ref	T	ref	ref	ref	ref	ref	ATM	UTR_3_PRIME: 548 bases from CDS
11	108237662	T	C	SNV	T/C	ref	T/C	ref	ref	ref	ref	ref	ref	ref	ref	ATM	UTR_3_PRIME: 1427 bases from CDS
11	108238183	C	T	SNV	ref	ref	ref	ref	ref	C/T	ref	ref	ref	ref	ref	ATM	UTR_3_PRIME: 1948 bases from CDS
11	108238434	C	G	SNV	C/G	ref	C/G	ref	ref	ref	ref	ref	ref	ref	ref	ATM	UTR_3_PRIME: 2199 bases from CDS
11	108238455	A	G	SNV	A/G	ref	A/G	ref	ref	ref	ref	ref	ref	ref	ref	ATM	UTR_3_PRIME: 2220 bases from CDS
11	108239628	G	T	SNV	G/T	ref	G/T	T	ref	T	ref	ref	ref	ref	ref	ATM	UTR_3_PRIME: 3393 bases from CDS
11	108239648	T	C	SNV	T/C	C	T/C	ref	ref	ref	ref	ref	ref	ref	ref	ATM	UTR_3_PRIME: 3413 bases from CDS

Reads from each sample were aligned to the hs37d5 sequence (hg19 and decoy sequences) using the Burrows-Wheeler Aligner 0.6.2. The whole exome analysis detected the homozygous mutation at a splice donor site of the *ATM* gene (chr11:108164206, IVS31+2T>A).
Reference: hs37d5 sequence (hg19 and decoy sequence).
Type, SNV: Single nucleotide variation, DEL: Deletion.
Genotype. C1, C2, C3, C4, C5 and C6: Healthy control, AT1OS: parental cells, 262: ATiPS-262 cells, 263: ATiPS-263 cells, 264: ATiPS-264 cells, 024: ATiPS-024 cells.

Supplemental Table S4. Primer sets for quantitative RT-PCR		
**Gene Product**	**Forward (5'→3')**	**Reverse (5'→3')**
GAPDH	GCTCAGACACCATGGGGAAGGT	GTGGTGCAGGAGGCATTGCTGA
OCT3/4	CGAGCAATTTGCCAAGCTCCTGA	TTCGGGCACTGCAGGAACAAATTC
SOX2	CATGGACAGTTACGCGCACATGA	TGGTAGTGCTGGGACATGTGAAGT
NANOG	TCCAGCAGATGCAAGAACTCTCC	TCCAGGCCTGATTGTTCCAGGATT
KLF4	AATTACCCATCCTTCCTGCCC	GTAATCACAAGTGTGGGTGGC
c-MYC	AAGACTCCAGCGCCTTCTCTC	AGGAGCCTGCCTCTTTTCCAC
TERT	GAGCAAGTTGCAAAGCATTG	TTTCTCTGCGGAACGTTCTG
DNMT3B	AATCCTGGAGGCTATCCGCAC	GTCAGAGCCATCCCCATCTTC
SOX1	CAATGCGGGGAGGAGAAGTC	CTCTGGACCAAACTGTGGCG
PAX6	AATAACCTGCCTATGCAACCC	AACTTGAACTGGAACTGACACAC
NESTIN	CAGCTGGCGCACCTCAAGATG	AGGGAAGTTGGGCTCAGGACTGG

Supplemental Table S5. Antibodies for immunocytochemistry			
**1st Ab**			**Dilution**
Oct3/4 (C-10)	mouse monoclonal IgG2b	Santa Cruz / sc-5279	1:300
Sox2	rabbit polyclonal	Millipore / AB5603	1:200
Nanog	rabbit polyclonal	Reprocell / RCAB0003P	1:200
SSEA4(MC-813-70)	mouse monoclonal IgG3	Millipore /MAB4304	1:300
Tra-1-60(TRA-1-60)	mouse monoclonal IgM	Millipore /MAB4360	1:300
Sox1(EPR4766)	rabbit polyclonal IgG	Epitomics / 3510-1	1:100
Pax6	rabbit polyclonal	Abcam / ab5790	1:100
Nestin	rabbit polyclonal	Signa / N5413	1:100
ATM(Y170)	rabbit polyclonal IgG	Abcom / ab32420	1:500
P1981ATM(7C10D8)	mouse monoclonal IgG2ak	Rockland / 200-301-500	1:500
**2nd Ab**			**Dilution**
Alexa Fluor 546 goat anti-mouse IgG(H+L)		Invitrogen / A11003	1:300
Alexa Fluor 488 F(ab')2 fragment of goat anti-mouse IgG(H+L)		Invitrogen / A11017	1:300
Alexa Fluor 546 goat anti-rabbit IgG(H+L)		Invitrogen / A11010	1:300
Alexa Fluor 488 goat anti-rabbit IgG(H+L)		Invitrogen / A11008	1:300
